# Regulation of CHD2 expression by the *Chaserr* long noncoding RNA gene is essential for viability

**DOI:** 10.1038/s41467-019-13075-8

**Published:** 2019-11-08

**Authors:** Aviv Rom, Liliya Melamed, Noa Gil, Micah Jonathan Goldrich, Rotem Kadir, Matan Golan, Inbal Biton, Rotem Ben-Tov Perry, Igor Ulitsky

**Affiliations:** 1 Department of Biological Regulation, Weizmann Institute of Science, Rehovot, Israel; 2 National Institute for Biotechnology in the Negev and Department of Microbiology, Immunology and Genetics, Ben-Gurion University of the Negev, Beer-Sheva, Israel; 3 Department of Molecular Cell Biology, Weizmann Institute of Science, Rehovot, Israel; 4 Department of Veterinary Resources, Weizmann Institute of Science, Rehovot, Israel

**Keywords:** Gene regulation, Chromatin remodelling, Long non-coding RNAs

## Abstract

Chromodomain helicase DNA binding protein 2 (*Chd2*) is a chromatin remodeller implicated in neurological disease. Here we show that *Chaserr*, a highly conserved long noncoding RNA transcribed from a region near the transcription start site of *Chd2* and on the same strand, acts in concert with the CHD2 protein to maintain proper *Chd2* expression levels. Loss of *Chaserr* in mice leads to early postnatal lethality in homozygous mice, and severe growth retardation in heterozygotes. Mechanistically, loss of *Chaserr* leads to substantially increased *Chd2* mRNA and protein levels, which in turn lead to transcriptional interference by inhibiting promoters found downstream of highly expressed genes. We further show that *Chaserr* production represses *Chd2* expression solely in *cis*, and that the phenotypic consequences of *Chaserr* loss are rescued when *Chd2* is perturbed as well. Targeting *Chaserr* is thus a potential strategy for increasing CHD2 levels in haploinsufficient individuals.

## Abstract (web summary)

The conserved long noncoding RNA *Chaserr* is transcribed upstream of the chromatin remodeler *Chd2*. Here, using mouse genetics and high throughput assays, the authors show that *Chaserr* inhibits expression of *Chd2* in *cis* and is required for postnatal mouse development.

## Introduction

The mammalian transcriptome is highly complex, and contains tens of thousands of noncoding RNA genes^1–4^. A significant subset of these, referred to as long noncoding RNAs (lncRNAs), are at least 200 nucleotides (nt) in length, 3′ polyadenylated, and 5′ capped, and are therefore structurally similar to mRNAs, but lack protein-coding potential^5^. Only a small portion of lncRNAs have been functionally characterized, and only very few of these have been studied in the context of organismal development^6^.

Chromodomain Helicase DNA Binding Protein 2 (*Chd2*) gene encodes an ATP-dependent chromatin-remodeling enzyme, which together with CHD1 belongs to subfamily I of the chromodomain helicase DNA-binding (CHD) protein family. Members of this subfamily are characterized by two chromodomains located in the N-terminal region and a centrally located SNF2-like ATPase domain^7^, and facilitate disassembly, eviction, sliding, and spacing of nucleosomes^8^. There are conflicting reports on the genomic occupancy of CHD2. According to one report, based on ChIP-seq data obtained with antibodies against CHD1 and CHD2, both proteins bind predominantly in the proximity of gene promoters and share up to 60% of their DNA-binding sites in human cell lines^9^. Another study used MNase-ChIP-seq of endogenously tagged *Chd1* and *Chd2* in mouse embryonic stem cells (mESCs)^10^, and reported that the two proteins have different binding patterns—CHD1 binds predominantly to promoter regions, whereas CHD2 is associated with gene bodies of actively transcribed genes. CHD2 has also been linked to the deposition and incorporation of the H3.3 histone variant at transcriptionally active  genes^9,11,12^ and at DNA damage sites, with the latter activity promoting repair of double-strand breaks^13^.

Mice homozygous for a gene-trap stop cassette in intron 27 of *Chd2* survive until E18.5 with a marked growth retardation, and no viable offspring of these mice can be recovered^14^. Heterozygotes with this mutation show increased postnatal mortality at days 1-4, and in the long term they exhibit growth retardation, shorter life spans, and altered morphology in various organs. However, a dominant negative effect of the truncated protein could not be excluded in this model. A different model for *Chd2* loss of function was recently created by the International Mouse Phenotyping Consortium, where exon 3 was replaced by a lacZ cassette and a stop signal^15^. No significant changes in mortality and aging were reported for these mice, but they exhibit slightly decreased body weight and length, skeletal abnormalities, abnormal bone structure, decreased fat amount and bone mineral density, and abnormalities in blood composition, such as decreased erythrocyte cell number, hemoglobin content, and mean platelet volume (http://www.mousephenotype.org/).

In humans, *CHD2* haploinsufficiency is associated with neurodevelopmental delay, intellectual disability, epilepsy, and behavioral problems (reviewed in ref. ^16^). Studies in mouse models and cell lines also implicate *Chd2* in neuronal dysfunction: perturbations of *Chd2* affect neurogenesis in the mouse developing the cerebral cortex^17^ and in human stem cells differentiated to neurons^18^, and loss of a single *Chd2* copy leads to deficits in neuron proliferation and a shift in neuronal excitability^19^. Therefore, approaches for increasing CHD2 levels may have therapeutic relevance.

Multiple lines of evidence point to a strong link between lncRNA functions and those of chromatin-modifying complexes^20,21^. Numerous chromatin modifiers have been reported to interact with lncRNAs^20^. In addition, lncRNAs in vertebrate genomes are enriched in the vicinity of genes that encode for transcription-related factors^2,22^, including numerous chromatin-associated proteins, but the functions of the vast majority of these lncRNAs remain unknown. We hypothesized that the proximity of some lncRNA genes to genes involved in chromatin biology may imply a functional connection. To explore the biology of such interactions, we focus on one of the most conserved lncRNAs in vertebrates, found in close proximity to *Chd2*.

## Results

### *Chaserr* is a conserved lncRNA located upstream of *Chd2*

**Fig. 1:**
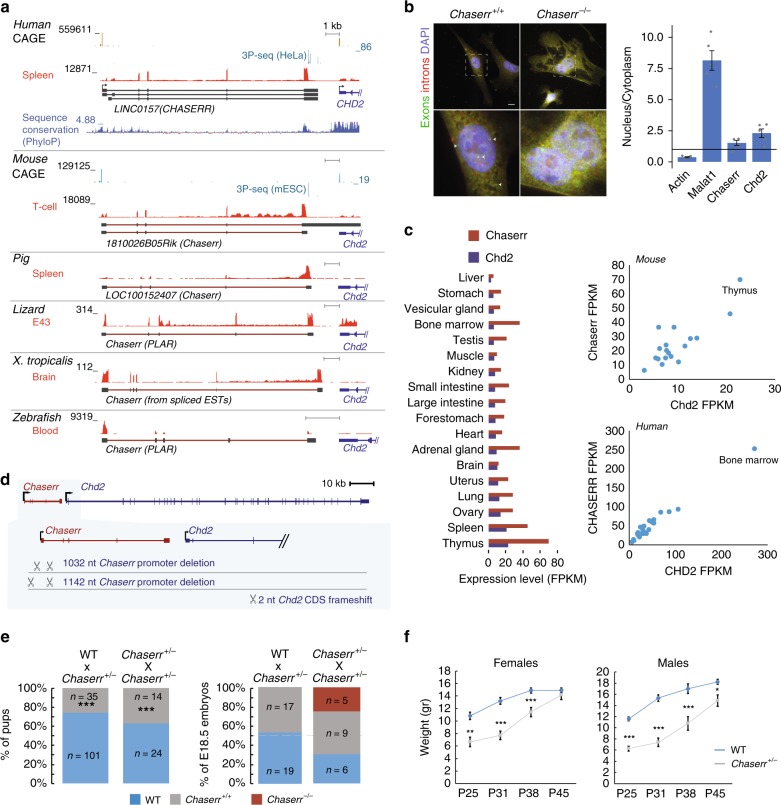
*Chaserr* is essential for postnatal viability. **a** Genomic locus of *Chaserr* in the indicated vertebrate genomes. CAGE read coverage is from the FANTOM5 project^75^. 3P-seq data are from ref. ^76^. RNA-seq data are from HPA^77^, ENCODE (mouse), FAANG (pig), SRP009831 (lizard), SRP039546 (xenopus), and SRP024369 (zebrafish). **b** Left: single-molecule FISH with probes targeting *Chaserr* introns (red) and exons (green) in mouse embryonic fibroblasts. *Chaserr* exons are marked by white arrowhead. Scale bar shows 10 µm. Right: qRT-PCR comparing cytoplasm and nuclear fractions in mESCs. *n* = 5. **c** Left: *Chaserr* and *Chd2* RNA expression across different tissues profiled in the mouse BodyMap^78^. Right: scatter plots for *Chaserr* and *Chd2* expression in mouse BodyMap and human HPA^77^ projects. **d** Schematic of the positions of regions targeted by gRNAs used to generate *Chaserr*^−/−^ and *Chd2*^m/m^ mice. **e** Left: neonate survival rates for the indicated crossing. Right: embryonic survival rates at the indicated embryonic time points. **f** Weights of neonates at the indicated age (males *n* = 7–15, females *n* = 6–18 per group). Error bars show S.E.M. *P < 0.05, **P < 0.01, and ***P < 0.001 (**e** chi-squared test. **f** two-sided *t* test). Source data are provided as a Source Data file

*1810026B05Rik* in mouse (which we denote as *Chaserr*, for *CHD2 adjacent, suppressive regulatory RNA*) and *LINC01578*/*LOC100507217* in human (*CHASERR*), are almost completely uncharacterized lncRNAs, found upstream of and transcribed from the same strand as *Chd2* (Fig. 1a). *Chaserr* has five exons, is polyadenylated, and is a bona fide lncRNA according to PhyloCSF^23^ (Supplementary Fig. 1a), CPAT^24^ (coding probability 0.296), and CPC^25^ (coding potential score −1.23). According to FANTOM5 transcription start site (TSS) annotations, 3P-seq poly(A) site mapping, and RNA-seq data, *Chaserr* transcript is independent of *Chd2* (Fig. 1a). The tandem organization with *Chd2*, *Chaserr* exon–intron structure, and parts of *Chaserr* sequence are conserved throughout vertebrates (Fig. 1a), which makes it one of the most conserved mammalian lncRNAs^22,26^. According to RefSeq annotation, the last exon of *Chaserr* in mouse overlaps *Chd2*; however, according to RNA-seq and 3P-seq data from various tissues, and to 3′ RACE in mouse embryonic fibroblasts (mEFs) (Supplementary Fig. 1b), the predominant *Chaserr* isoform ends ~500 bp after its last 3′ splice site, ~2.2 kb upstream of the *Chd2* TSS (as in GENCODE transcript ENSMUST00000184554), and we therefore considered this isoform in further studies. By using single-molecule RNA FISH, we found that the RNA product of *Chaserr* is mostly localized in the nucleus, in proximity to the *Chd2* site of transcription, and subcellular fractionation shows that it is enriched in the chromatin fraction (Fig. 1b and Supplementary Fig. 1c). This nuclear enrichment is due at least in part to nonsense-mediated decay (NMD) that acts on *Chaserr*, likely triggered by a 117-codon non-conserved ORF that ends in the second exon (Supplementary Fig. 1d, e).

*Chaserr* and *Chd2* mRNA are tightly co-expressed across a panel of human and mouse tissues, and during mouse development according to ENCODE and FANTOM5 data (Fig. 1c and Supplementary Fig. 1f, g). Interestingly, both genes are expressed at appreciable levels in all studied samples, with particularly high expression in lymphocytes. The ratio between the expression levels of the two genes is also similar across the tissues, with notable exceptions of neuronal cells and fibroblasts, where *Chaserr* levels are relatively high (Fig. 1c and Supplementary Fig. 1f, g).

### *Chaserr* is required for postnatal survival

In order to study its function, we generated *Chaserr* null alleles in mice by injection into fertilized oocytes of CRISPR/Cas9 mRNA with a pair of gRNAs targeting sequences flanking the promoter and the first exon of *Chaserr* (Fig. 1d). This resulted in two different *Chaserr*^+/–^ mouse strains that carry deletions of 1032 and 1142 bps (Supplementary Fig. 1h), which were sufficient to abolish expression of mature *Chaserr* (see below), and the two lines were phenotypically indistinguishable from each other, and so we used them interchangeably in subsequent experiments. Strikingly, out of 38 pups born following crosses between *Chaserr*^+/–^ mice, we observed no *Chaserr*^–/–^ pups. The numbers of *Chaserr*^+/–^ pups at weaning also deviated from expected Mendelian ratios (~37%, *P* < 10^–8^, Fig. 1e), and the surviving mice exhibited substantial growth retardation, occasional malocclusion, and neonatal lethality (Fig. 1f and Supplementary Fig. 1i–k). Pathological analysis of *Chaserr*^+/–^ mice revealed a wide range of abnormalities, including fat depletion, kyphosis, and thymic depletion, neither of which was highly penetrant. Out of 136 pups born from *Chaserr*^+/–^ and Chaserr^+/+^ crosses, 35 were *Chaserr*^+/–^ (~25%), significantly less than the expected 50% (*P* < 10^−5^, Fig. 1e), suggesting that two copies of *Chaserr* are required for proper postnatal survival. In contrast, embryos were recovered with the expected Mendelian ratios in crosses between *Chaserr*^+/–^ and *Chaserr*^+/+^ mice and in *Chaserr*^+/–^ intercrosses (Fig. 1e and Supplementary Fig. 1l), suggesting that *Chaserr* is required for postnatal, but not embryonic, survival. *Chaserr*^+/–^ females very rarely became pregnant, and therefore we focused on crosses between *Chaserr*^+/+^ females and *Chaserr*^+/–^ males for most of this study.

**Fig. 2:**
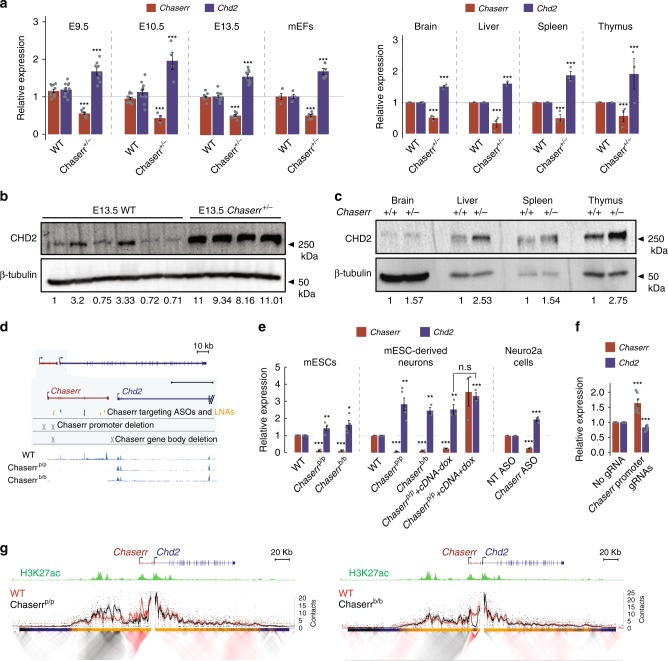
*Chaserr* represses *Chd2* expression. **a** qPCR of the indicated genes in whole embryos from the indicated developmental stage, mEFs, or adult tissues with the indicated background. *n* for each group of embryos or mEFs is indicated in parentheses. Normalized to *Actb*. *n* = 3 for the tissues. **b** Western blot for the indicated protein in individual E13.5 whole embryos from indicated background. **c** As in **b** for the adult tissues. **d** Top: scheme of regions targeted by gRNAs used to generate *Chaserr*^p/p^, *Chaserr*^b/b^ mESCs, or the antisense reagents; Bottom: RNA-seq read coverage in mESCs from the indicated background. **e**, **f** qRT-PCR of the indicated genes in the indicated backgrounds or treatments. Normalized to *Actb*. *n* = 3–5. **g** Targeted Chromosome Conformation Capture (4C) analysis with the *Chd2* promoter as the viewpoint in WT vs. *Chaserr*^p/p^ (left) or WT vs. *Chaserr*^b/b^ (right) mESCs. Top to bottom: scheme of the *Chd2*-*Chaserr* locus; H3K27ac ChIP-seq signal in mESCs; smoothed trend lines and raw counts of the contact profiles in the indicated cell lines; domainogram^79^ showing mean contact per fragment end for a series of window sizes. Ratio quantifications are shown below Western blots. Source data are provided as a Source Data file. Error bars show S.E.M. *P < 0.05, **P < 0.01, and ***P < 0.001 (**a**, **e**, **f**: two-sided *t* test)

Due to the close proximity and co-expression of *Chaserr* and *Chd2*, we tested whether *Chd2* expression is affected in *Chaserr*^+/–^ embryos. *Chd2* mRNA was significantly upregulated by ~1.5-fold during embryonic development at the examined time points (E9.5, E10.5, and E13.5), in mEFs derived from E13.5 embryos, and in four adult tissues (Fig. 2a). Western blot (WB) in E13.5 embryos and in the adult tissues also demonstrated strong upregulation of CHD2 protein (Fig. 2b, c).

### *Chaserr* RNA or its transcription repress *Chd2*

As *Chaserr*^–/–^ mice could not be generated, in order to study the regulation of *Chd2* in *Chaserr*^–/–^ cells and efficiently compare different *Chaserr* perturbations, we used CRISPR/Cas9 in mouse embryonic stem cells (mESCs) to engineer clones with either homozygous loss of *Chaserr* promoter and the first exon (*Chaserr*^p/p^), or deletion of the rest of the *Chaserr* gene body from the first intron to just downstream of *Chaserr* polyadenylation site (*Chaserr*^b/b^) (Fig. 2d). Importantly, the *Chaserr*^b/b^ line has an intact *Chaserr* promoter, which still recruits RNA Pol2 (Supplementary Fig. 2a). *Chaserr* expression was abolished in both mutant lines, as evident in RNA-seq data (Fig. 2d), leading to significant *Chd2* mRNA upregulation (Fig. 2e). This upregulation is consistent with a previous study that examined *Chaserr* as part of a panel of lncRNAs (*Chaserr* was referred to as linc2025 in that study) and also observed an increase in *Chd2* upon deletion of *Chaserr* promoter^27^. *Chd2* was also significantly upregulated in neurons derived from *Chaserr*^p/p^ and *Chaserr*^b/b^ mESCs (Fig. 2e). Similarly, targeting of *Chaserr* in Neuro2a cells by using antisense oligonucleotides (ASOs) or LNA Gapmers reduced *Chaserr* expression by ~75%, and led to a significant increase in *Chd2* mRNA and protein levels (Fig. 2e and Supplementary Fig. 2b, c). Furthermore, induction of *Chaserr* expression from the endogenous locus by using CRISPR activation^28^ resulted in a decrease in *Chd2* mRNA levels (Fig. 2f). We note that other perturbation methods, like insertion of polyA sites^29^ are not effective for perturbing *Chaserr*^27^. We conclude that an intact *Chaserr* represses *Chd2* expression in all the systems we studied.

Importantly, *Chaserr*^b/b^ mESCs have an intact *Chaserr* promoter, which recruits RNA Pol2 (Supplementary Fig. 2a), and thus this line helps address the possibility of a competition between the *Chaserr* and *Chd2* promoters over shared enhancers. Indeed, Targeted Chromosome Conformation Capture (4C) analysis of the two lines showed that while deletion of *Chaserr* promoter resulted in a pronounced increase of contacts between the *Chd2* promoter and an array of enhancers upstream of the *Chaserr*/*Chd2* locus, no substantial changes were observed in the *Chaserr*^b/b^ cells (Fig. 2g), suggesting that enhancer competition is not the predominant cause of the increased *Chd2* levels in these cell lines.

In order to test whether the mature *Chaserr* RNA is sufficient for repression of *Chd2*, we infected the *Chaserr*^p/p^ mESCs with a lentivirus carrying a doxycycline (dox)-inducible *Chaserr* cDNA. *Chaserr* was overexpressed relative to the WT levels by ~3.5-fold following Dox addition, but *Chd2* mRNA expression was not affected compared with no-Dox control (Fig. 2e). We conclude that either the RNA product of *Chaserr*, or transcription from its endogenous locus (which might be affected by the ASO/Gapmer cleavage of the nascent transcript), are required for repression of *Chd2* expression.

### Chaserr acts in *cis*

**Fig. 3:**
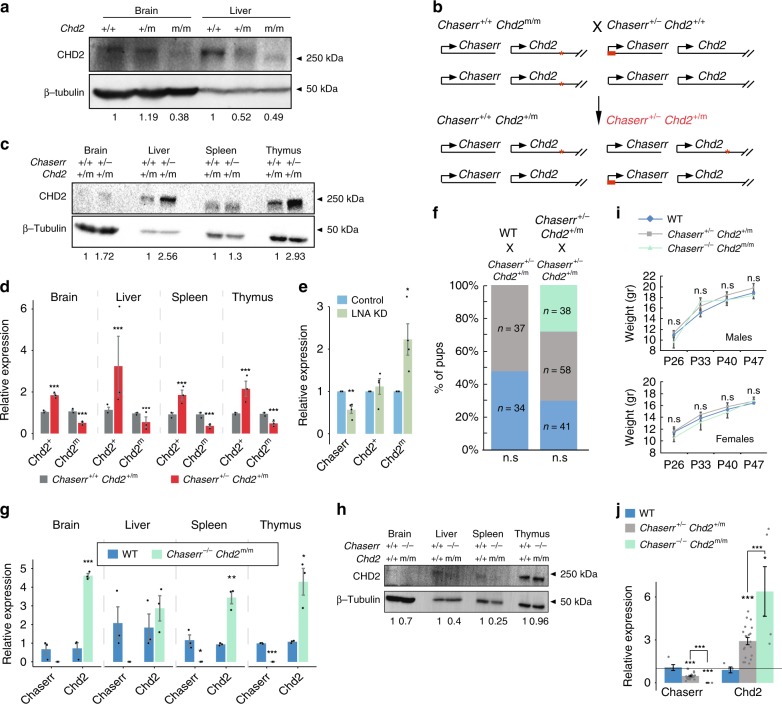
*Chaserr* regulates *Chd2* expression in *cis*. **a** CHD2 Western blot in the indicated tissues from mice with the indicated genotypes. **b** Scheme of the cross between *Chaserr*^+/−^ and *Chd2*^m/m^ mice. **c** Western blot in the indicated tissues from mice with the indicated genotypes. **d** *Chd2* allele-specific expression in the indicated backgrounds in four different mouse tissues. Normalized to *Actb*. *n* = 3 for the indicated tissues. **e** Changes in expression of *Chaserr*, or of *Chd2* mRNA from the indicated allele, in *Chaserr*^+/−^ *Chd2*^m/+^ mEFs transfected with a mix of two LNAs targeting *Chaserr* or non-targeting control (NT). **f** Neonate survival rates for the indicated crossing. **g** qRT-PCR in the indicated adult tissues and backgrounds. Normalized to *Actb*. *n* = 3 for the indicated tissues. **h** CHD2 Western blot from the indicated tissues and backgrounds. **i** Pup weights for the *Chaserr*^+/−^ *Chd2*^m/+^ intercross. *n* = 5–19 pups per genotype per time point. **j** qRT-PCR for mEFs with the indicated background. Normalized to *Actb*. WT *n* = 5, *Chaserr*^+/−^ *Chd2*^+/m^ *n* = 20, *Chaserr*^+/−^ *Chd2*^+/m^ *n* = 5. Error bars show S.E.M. *P < 0.05, **P < 0.01, and ***P < 0.001 (**d**, **e**, **g**, **i**, **j**: two-sided *t* test. **f**: chi-squared test). Ratio quantifications are shown below Western blots. Source data are provided as a Source Data file

We next examined whether the early lethality of mice with *Chaserr* loss of function is mediated by increased *Chd2* levels. We used CRISPR/Cas9 with a single gRNA targeted to the third exon of *Chd2* (Fig. 1d and Supplementary Fig. 2d) to generate *Chd2*^m/m^ mice that had a 2-bp deletion in the coding sequence of *Chd2*. This mutation had a mild effect on Chd2 mRNA expression, but dramatically reduced CHD2 protein levels, as expected from a frameshift in the main coding frame, with the residual protein possibly emanating from an alternative start codon (Fig. 3a, note that the monoclonal antibody we used recognizes a peptide in the C-terminal part of CHD2). *Chd2*^m/m^ mice were born at expected Mendelian ratios (Supplementary Fig. 2e), and similarly to the *Chd2*^tm1b(EUCOMM)Hmgu^ mice generated by insertion of a LacZ cassette into the second intron and deletion of the third exon of *Chd2*^30^, had mild reduction in size and in subcutaneous adipose tissue, but no gross developmental or fertility phenotypes. This enabled us to breed *Chaserr*^+/–^ mice with *Chd2*^m/m^ mice (Fig. 3b). We intercrossed the resulting *Chaserr*^+/*–*^ *Chd2*^m/+^ offspring, and out of 44 pups born from such crosses, only 14 (~32%) were *Chaserr*^*+/–*^ (Supplementary Fig. 2f), suggesting that one hypomorphic allele of *Chd2* is not sufficient for compensating for loss of one *Chaserr* allele. Indeed, CHD2 protein levels were substantially higher in *Chaserr*^+/–^ *Chd2*^m/+^ mice when compared with their *Chaserr*^+/+^ *Chd2*^m/+^ littermates (Fig. 3c), potentially explaining the neonatal lethality and growth retardation (Supplementary Fig. 2g).

The *Chaserr*^+/–^ *Chd2*^m/+^ offspring that survived allowed us to test with allele-specific qRT-PCR whether *Chaserr* affected *Chd2* expression from the *cis* allele in vivo (Supplementary Fig. 2h). Only *Chd2* in *cis* to the *Chaserr*^–^ allele was upregulated in *Chaserr*^+/–^ *Chd2*^m/+^ mice when compared with *Chaserr*^+/+^ *Chd2*^m/+^ littermates (Fig. 3d), suggesting that *Chaserr* represses *Chd2* through *cis*-acting regulation. Interestingly, the *Chd2* mRNA produced from the *Chaserr*^+^ allele was repressed compared with WT levels, hinting at a possible feedback regulation resulting from excess CHD2 expression (see Discussion). mEFs derived from E13.5 *Chaserr*^+/–^ *Chd2*^m/+^ embryos allowed us to test whether the effect of knocking down *Chaserr* on *Chd2* also occurs strictly in *cis*. Indeed, treatment of these mEFs with LNA Gapmers targeting *Chaserr* (which is expressed only from the *Chd*^m^ allele) resulted in an increase in expression of *Chd*^*m*^ mRNA, but did not affect *Chd*^+^ mRNA levels (Fig. 3e), further solidifying the *cis*-acting function of *Chaserr* RNA product or the act of its transcription.

### *Chd2* loss of function rescues phenotypes of *Chaserr*^*–/–*^ mice

In order to directly test whether the severe *Chaserr* loss-of-function phenotype is mediated by CHD2 overexpression, we used CRISPR/Cas9 to delete the *Chaserr* promoter and the first exon in a *Chd2*^m/m^ mouse (Supplementary Fig. 3a), and thus generated a model in which loss of *Chaserr* increases the expression of a hypomorphic allele of *Chd2*. Out of 137 pups born from intercrosses of *Chaserr*^+/–^ *Chd2*^+/m^ mice, 38 were *Chaserr*^–/–^ *Chd2*^m/m^ (~27%), 58 *Chaserr*^+/–^ *Chd2*^+/m^(~42%), and 41 WT (~30%), which did not deviate significantly from normal Mendelian ratios (P = 0.2; Fig. 3f and Supplementary Fig. 3b, c), despite up to sixfold upregulation of *Chd2* mRNA in different tissues of *Chaserr*^–/–^ *Chd2*^m/m^ mice (Fig. 3g). Despite *Chd2* mRNA upregulation, CHD2 mutant protein levels in tissues from *Chaserr*^–/–^ *Chd2*^m/m^ mice were not higher than the WT protein levels in WT mice (Fig. 3h). *Chaserr*^–/–^ *Chd2*^m/m^ and *Chaserr*^+/–^ *Chd2*^+/m^ mice showed no significant differences in weight (Fig. 3i and Supplementary Fig. 3d), and no obvious phenotypes, in stark contrast to the common and pleiotropic phenotypes observed in *Chaserr*^+/–^ neonates on *Chd2*^+/+^ background. A hypomorphic allele of *Chd2* can thus rescue the lethality caused by loss of *Chaserr*, when the two occur on the same allele.

To further characterize the changes in levels of *Chd2* mRNA in the double mutants, we isolated mEFs from E13.5 embryos from *Chaserr*^+/+^, *Chaserr*^+/–^, and *Chaserr*^–/–^ genotypes on *Chd2*^m/m^ background. qRT-PCR analysis showed that *Chaserr* expression is completely abolished in *Chaserr*^–/–^ *Chd2*^m/m^ mice, and that *Chd2* mRNA was significantly overexpressed in a *Chaserr* dosage- dependent manner (Fig. 3j).

### *Chaserr* loss leads to extensive transcriptional changes

**Fig. 4:**
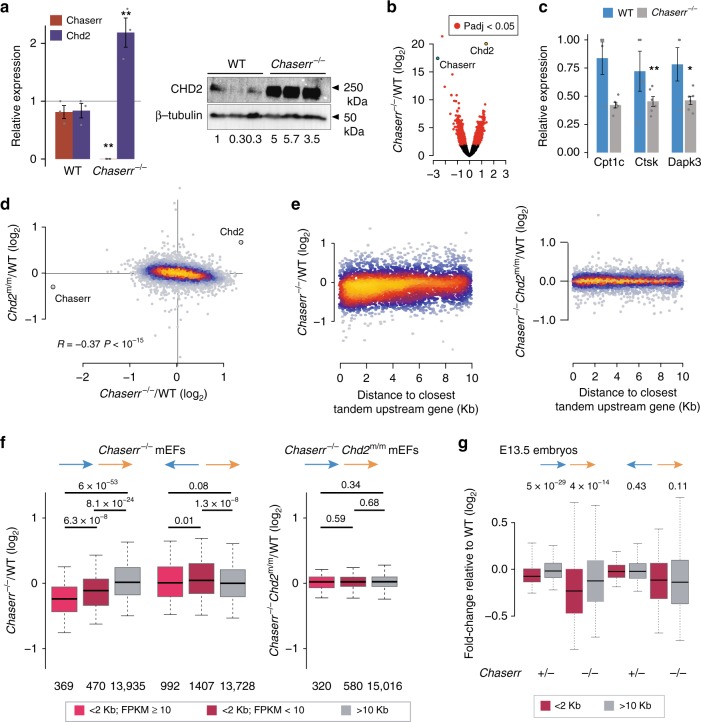
Transcriptional dysregulation following loss of *Chaserr*. **a** qRT-PCR (left) and Western blot (right) of *Chd2* expression in mEFs from the indicated background. *n* = 3. qRT-PCR normalized to *Actb*. Western blot shows mEFs from three embryos for each background. Error bars show S.E.M. **P < 0.01 (two-sided *t* test). **b** Volcano plots for the indicated differential expression comparison by using DESeq2^62^. **c** qRT-PCR of the indicated genes in WT and *Chaserr*^−/−^ mEFs. Normalized to *Actb*. WT *n* = 4, *Chaserr*^−/−^ *n* = 6 *P < 0.05, **P < 0.01 (two-sided *t* test). **d** Changes in gene expression in *Chaserr*^−/−^ and *Chd2*^m/m^ mEFs. Correlation computed by using Spearman’s correlation. Color indicates point density. **e** Change in expression in *Chaserr*^−/−^ or *Chaserr*^−/−^ *Chd2*^m/m^ vs. WT mEFs and distance to the 3′ end of the closest upstream gene transcribed from the same strand, for genes for which this distance is <10 kb. Color indicates point density. **f** Changes in gene expression in *Chaserr*^−/−^ or *Chaserr*^−/−^ *Chd2*^m/m^ vs. WT mEFs for genes with the indicated distances from the closest upstream gene. Genes with distance <2 kb were further subdivided based on the expression levels of the upstream gene. The number of genes in each group is indicated below the distance threshold. Boxplots show the 5th, 25th, 50th, 75th, and 95th percentiles. **g** As in **f**, for the E13.5 whole embryo RNA-seq. *P*-values computed by using Wilcoxon rank-sum test. Ratio quantifications are shown below Western blots. Source data are provided as a Source Data file. Boxplots show the 5th, 25th, 50th, 75th, and 95th percentiles

Having established that the severe phenotype resulting from *Chaserr* loss is mediated by CHD2, we were next interested to understand the consequences on gene expression of *Chaserr* loss and CHD2 upregulation. Beyond the increase in *Chd2* mRNA levels, limited changes in gene expression were observed in *Chaserr*^+/–^ E9.5 or E13.5 embryos, mEFs, or brains (Supplementary Fig. 4a). In contrast, hundreds of genes were differentially expressed in *Chaserr*^–/–^ mEFs, which showed a more than two-fold increase in *Chd2* mRNA and protein levels (Fig. 4a). Changes in gene expression in three independently-derived *Chaserr*^–/–^ mEFs were strongly correlated with those observed in an E13.5 *Chaserr*^–/–^ embryo (*R* = 0.4, *P* < 10^−15^). The 1493 significantly downregulated genes (down by at least 25%, adjusted *P* < 0.05) were enriched for various GO categories, such as nervous and connective tissue development and transcriptional regulation, and were very significantly enriched for genes whose loss of function in mice is associated with phenotypes such as decreased length of long bones, decreased body weight, respiratory distress, and premature death (Supplementary Data 1), closely related to the phenotypes observed in our *Chaserr* loss-of-function model. Specifically, some of the most downregulated genes, such as *Ctsk*, *Cpt1c*, and *Dapk3*, which were also validated by qRT-PCR (Fig. 4c), are known to be required for proper embryonic development^31^. In contrast, the 616 significantly upregulated genes (up by at least 25%, adjusted *P* < 0.05) were enriched for various RNA-related GO categories (Supplementary Data 1).

Gene expression changes in *Chaserr*^–/–^ mEFs were significantly inversely correlated with those in *Chd2*^m/m^ mEFs, where *Chaserr* was downregulated and *Chd2* was upregulated (Fig. 4d and Supplementary Fig. 4b). We also profiled *Chaserr*^–/–^ *Chd2*^m/m^ mEFs, and found no correlation between the changes observed between these and WT mEFs, and the changes in *Chaserr*^–/–^ mEFs (Spearman *R* = −0.003, *P* = 0.68). Instead, changes in *Chaserr*^–/–^ *Chd2*^m/m^ mEFs were similar to those in *Chd2*^m/m^ mEFs (Supplementary Fig. 4b). The correlations between expression changes should be interpreted with caution as they are derived from different crosses, and yet they support the notion that the downstream effects of loss of *Chaserr* are driven by CHD2 upregulation.

### *Chaserr* loss leads to transcriptional interference between close genes

Inspection of the loci of some of the most downregulated genes in *Chaserr*^–/–^ mEFs, such as *Ctsk* and *Dapk3* (Fig. 4c), led us to suspect that promoters of some of the downregulated genes might be preferentially found in close proximity to transcription termination sites (TTSs) of genes transcribed on the same strand (and thus potentially susceptible to transcriptional interference), in a similar organization to the *Chaserr*–*Chd2* locus. Indeed, we found a mild yet highly significant correlation between changes in gene expression in *Chaserr*^–/–^ mEFs and the distance to the TTS of the closest tandem upstream gene (Spearman *R* = 0.2, *P* < 2 × 10^−15^), with the effect observed mostly when the distance was shorter than ~6 kb (Fig. 4e). In contrast, no such effect was observed in *Chaserr*^–/–^ *Chd2*^m/m^ mEFs (*R* = 0.0055, *P* = 0.45, Fig. 4e). Further, the downregulation was stronger when the expression of those upstream neighboring genes was higher (Spearman *R* = −0.17, *P* = 3.4 × 10^−7^ between the change in expression of the downstream gene and the absolute expression of the upstream gene for intergenic distances < 2 kb). Genes with a close and abundant upstream neighbor were significantly repressed in *Chaserr*^–/–^ mEFs, and in stark contrast, much smaller differences, and in the opposite direction, were observed for neighboring genes transcribed on opposite strands, when considering distances between 5′ ends (Fig. 4f). Similar trends were also found in *Chaserr*^+/–^ and *Chaserr*^–/–^ E13.5 embryos (Fig. 4g).

**Fig. 5:**
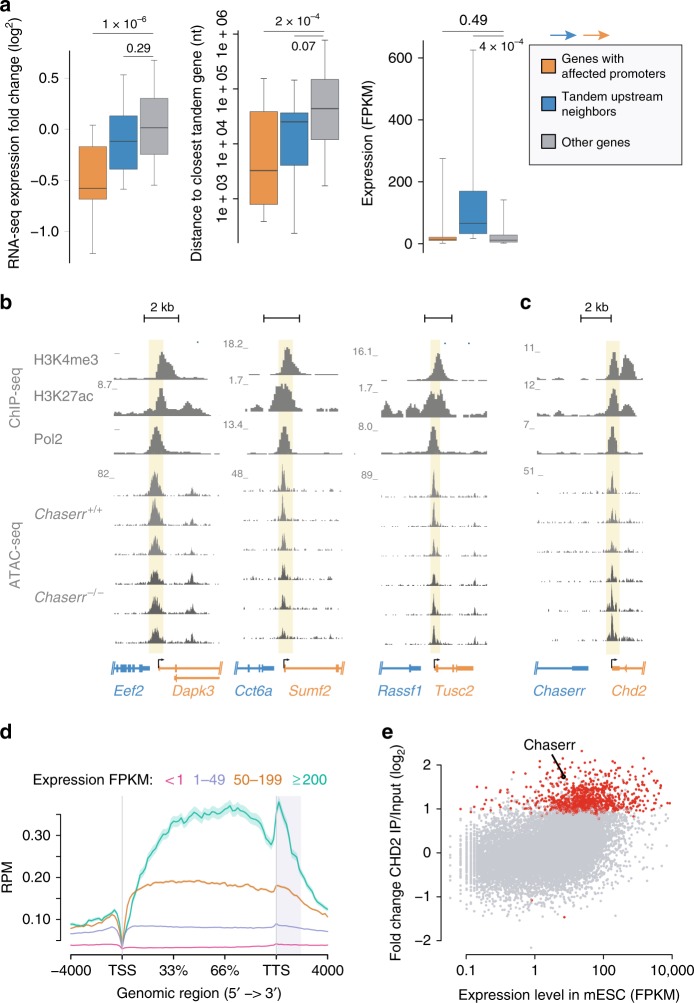
Changes in chromatin accessibility following loss of *Chaserr*. **a** Changes in gene expression in *Chaserr*^−/−^ mEFs, distance to the closest upstream gene transcribed from the same strand, and the expression levels, for genes assigned to promoter peaks with reduced accessibility in *Chaserr*^−/−^ mEFs, their upstream neighbors on the same strand, and all other genes. Only genes with ATAC-seq peaks at their promoters were considered. *P*-values computed with two-sided Wilcoxon rank-sum test. Boxplots show the 5th, 25th, 50th, 75th, and 95th percentiles. **b** Examples of loci of genes with reduced accessibility in their core promoters in the *Chaserr*^−/−^ background. Top: ChIP-seq coverage in mEFs from the ENCODE project. Bottom: read coverage in ATAC-seq data from mEFs isolated from three embryos from each background. **c** As in **b**, for the *Chaserr*/*Chd2* locus. **d** Metagene of CHD2 occupancy^10^ on genes in mESCs, divided into four groups based on their expression FPKM. **e** Enrichment of CHD2 occupancy in mESCs in the 2-kb region downstream of the TTS when compared with input, as a function of mESC gene expression levels. Genes with adjusted *P* < 0.05 are in red. Source data are provided as a Source Data file

To further characterize the regulatory dysregulation, we performed ATAC-seq^32^ on WT and *Chaserr*^–/–^ mEFs. Consistently with the increase in *Chd2* production, we observed a mild increase of ~7% in accessibility of the *Chd2* promoter in *Chaserr*^–/–^ mEFs. Comparing WT and *Chaserr*^–/–^ mEFs, we found 80 and 677 regions with increased or reduced accessibility, respectively (DESeq2 *P* < 0.05), with 6 induced and 24 reduced peaks occurring near TSSs. Promoter peaks whose accessibility was significantly decreased in *Chaserr*^–/–^ mEFs were associated with repressed genes in the RNA-seq data (Fig. 5a). Strikingly, these promoters were separated by short intergenic regions from TTSs of other genes on the same strand (Fig. 5a, b, median distance of 3.3 kb compared with 43.8 kb for unaffected promoters). Notably, the upstream genes were expressed at dramatically higher levels than the affected genes or other genes (17-fold difference between median expression, Fig. 5a), and their expression was not substantially affected by loss of *Chaserr* (Fig. 5a). Promoters of 96 additional genes that showed reduction of >25% in *Chaserr*^–/–^ mEFs in the RNA-seq data and had an upstream neighbor on the same strand within <2 kb, had reduced ATAC-seq signal in *Chaserr*^–/–^ mEFs when considered as a group (*P* = 0.014, Wilcoxon two-sided test). Altogether, transcriptional interference from highly expressed and close upstream neighbors reduces promoter accessibility and gene expression of over 100 genes in *Chaserr*^–/–^ mEFs.

To test if these effects stem from CHD2 overexpression, we used the same methodology to compare *Chaserr*^–/–^ *Chd2*^m/m^ mEFs with WT mEFs, and observed far fewer changes in accessibility (20 regions with decreased accessibility and 64 with increased accessibility, based on the same criteria as above, and only 3 decreased peaks in promoter regions), further supporting the notion that the transcriptional interference in *Chaserr*^–/–^ cells is driven by excess CHD2 levels.

Interestingly, the presence of a TTS closely upstream of the promoters most affected by loss of *Chaserr* (Fig. 5a, b) resembles the organization of the *Chaserr* and *Chd2* locus (Fig. 5c). By using tagged CHD2 MNase-ChIP-seq data from mESCs^10^, we observed significant occupancy of CHD2 protein in the regions downstream of TTSs of hundreds of highly expressed genes, including downstream of *Chaserr* TTS (Fig. 5d, e), suggesting that the effect seen on promoter accessibility and expression of genes found closely downstream of TTSs of highly expressed genes might be a direct result of increased CHD2 activity at intergenic regions. In summary, CHD2 appears to function in regions downstream of TTSs in WT cells, particularly at highly expressed genes, and the CHD2 hyperactivity caused by loss of *Chaserr* leads to repression of promoters found immediately downstream to those regions.

### *Chaserr* loss leads to reduction in Pol2 pausing

**Fig. 6:**
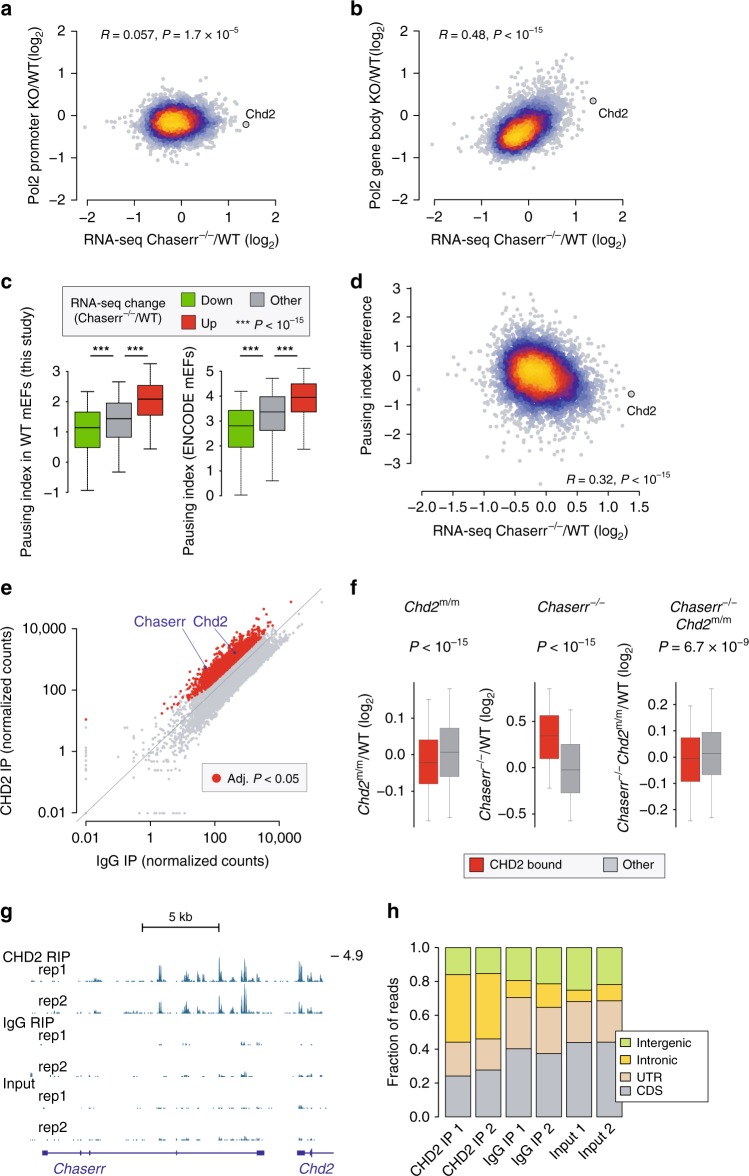
Changes in Pol2 occupancy in *Chaserr*^–/–^ mEFs and RNA binding by CHD2. **a** Correlation between changes in gene expression levels and changes in Pol2 occupancy at the promoter region (±300 bases around TSS). Color indicates point density. **b** As in **a**, but for changes in Pol2 occupancy at gene body regions (defined from 1 kb downstream of the TSS to the TTS). **c** Pol2 pausing index (see the “Methods” section) in our WT mEF Pol2 ChIP-seq data (left) and ENCODE mEF data (right) for genes with the indicated changes in gene expression in *Chaserr*^−/−^ mEFs (adjusted *P* < 0.05). Boxplots show the 5th, 25th, 50th, 75th, and 95th percentiles. **d** Changes in Pol2 pausing index as a function of changes in gene expression between *Chaserr*^−/−^ and WT mEFs. Color indicates point density. **e** Normalized RIP-seq read counts within gene bodies in CHD2 and IgG RIP libraries (average of two replicates). Red dots correspond to genes with adjusted *P* < 0.05 (DESeq2 analysis). **f** Changes in gene expression of genes significantly associated with CHD2 (from e) and other genes expressed in mEFs in the indicated RNA-seq-based comparison. Boxplots show the 5th, 25th, 50th, 75th, and 95th percentiles. **g** RIP-seq read coverage in the *Chaserr* locus. **h** Fractions of reads mapping to the indicated regions in the indicated RIP-seq library. Source data are provided as a Source Data file

An additional underlying factor contributing to dysregulation of gene expression emerged from ChIP-seq^33^ analysis of Pol2 in *Chaserr*^–/–^ and WT mEFs. We observed a significant correlation between changes in gene expression and changes in Pol2 occupancy at gene bodies, but not at promoters (Fig. 6a, b). This observation, together with the known roles of CHD1 in regulation of Pol2 pausing^34^, led us to suspect that Pol2 pausing might be affected by the increase in CHD2 levels caused by *Chaserr* loss. Indeed, genes upregulated in *Chaserr*^–/–^ mEFs were associated with a substantially higher pausing index in WT mEFs in our Pol2 ChIP-seq data, as well as in mEFs profiled by the ENCODE project (Fig. 6c); generally, increases in gene expression were correlated with reduction in Pol2 pausing at the affected genes (Fig. 6d). Overall, 400 (15.3%) of the genes significantly dysregulated in *Chaserr*^–/–^ mEFs were associated with a corresponding change of >25% in Pol2 pausing.

### CHD2 binds nascent RNAs and promotes gene expression

Several recent studies identified CHD2 as a noncanonical RNA-binding protein^35–37^. We therefore tested whether CHD2 might associate with *Chaserr* and with some of the RNAs deregulated by loss of *Chaserr* or of *Chd2*. Using RNA immunoprecipitation (RIP)^38^ followed by rRNA-depleted RNA sequencing (RIP-seq, see Methods), we found that CHD2 significantly associates with 2,427 RNAs, and that *Chaserr* is one of the most enriched RNAs in the IP compared with either the input or IgG controls (Fig. 6e and Supplementary Fig. 4c). Interestingly, CHD2 also substantially binds its own pre-mRNA (Fig. 6e). CHD2 RIP libraries contained a large number of intronic reads in the *Chaserr* locus (Fig. 6g) and in other loci (Fig. 6h and Supplementary Fig. 4d), suggesting that CHD2 predominantly binds nascent RNA. Strikingly, CHD2-bound RNAs were significantly upregulated in *Chaserr*^–/–^ mEFs, and conversely repressed relative to other genes in *Chd2*^m/m^ or *Chaserr*^–/–^ *Chd*^m/m^ mEFs (Fig. 6f). These data suggest that RNA binding by CHD2 typically promotes gene expression, including expression of the CHD2-bound *Chaserr* (which is repressed in *Chd2*^m/m^ mEFs, Fig. 4d), and thus *Chaserr* expression can act as a sensor of CHD2 levels.

## Discussion

Decoding lncRNA functions is a formidable challenge, in particular because of the substantial heterogeneity in their biology and modes of action. A prominent group of vertebrate lncRNAs, including some of the most conserved ones, are found in regions flanking genes involved in transcription, including numerous chromatin modifiers. We hypothesized that such co-localization may imply a connection between the biology of the chromatin modifier and the mode of action of the lncRNA, potentially through a feedback loop. By focusing on one such pair that is particularly highly conserved in evolution, we uncovered a lncRNA-mediated circuit that regulates the expression of *Chd2*.

Negative autoregulatory feedback loops are prevalent in RNA biology, and help tune levels of factors involved in mRNA splicing, polyadenylation, editing, and modifications, and in the processing of small RNAs^39–45^. There are also examples of such feedback loops regulating transcription-related genes^46^; however to the best of our knowledge, there are no known autoregulatory loops involving chromatin modifiers in mammals. We propose a model in which the *Chaserr*–*Chd2* tandem organization forms a negative feedback loop that tunes CHD2 levels. The genomic occupancy of CHD2 in regions immediately downstream of TTSs (Fig. 5d, e), combined with the correlation between changes in gene expression and changes in Pol2 occupancy in gene bodies in *Chaserr*^–/–^ mEFs (Fig. 6b), suggests that CHD2 acts at least partly within transcribed regions and in TTS-proximal regions. Accordingly, we show that under conditions of excess levels of CHD2 protein, this TTS-proximal occupancy is associated with transcriptional interference on downstream neighbors of highly expressed genes, especially when the intergenic regions between them are particularly short (Figs. 4e–g, 5a, b). Due to the short intergenic region between *Chaserr* and *Chd2*, we suggest that such transcriptional interference serves as the basis of a negative feedback loop that leads to repression of *Chd2* production when CHD2 protein levels are high, thus maintaining a tight control on its expression. In cells with *Chaserr* loss of function, this loop is compromised, and CHD2 is upregulated. Supporting this model, in *Chaserr*^+/–^ *Chd2*^m/+^ tissues, increase in CHD2 protein leads to repression of transcription of the *Chd2*^m^ allele, which is found next to an intact *Chaserr* (Fig. 3d). Furthermore, reduction in CHD2 protein in *Chd2*^m/m^ mEFs leads to a decrease in *Chaserr* levels and an increase in *Chd2* mRNA levels (Fig. 4d). Last, we found that CHD2 protein associates with the *Chaserr* RNA (Fig. 6e), supporting the possibility that excess CHD2 levels can be sensed by the *Chaserr* locus.

The exact mechanism by which *Chaserr* represses *Chd2* expression remains unknown. The simplest feedback model suggests that *Chaserr* transcription, or perhaps its termination and cleavage and polyadenylation, are important. Such a mechanism would resemble the activity of the SRG1 noncoding RNA in the yeast *S. cerevisiae* that represses the transcription of SER3, which is found immediately downstream of SRG1 and on the same strand, by deposition of nucleosomes at the SER3 promoter that prevent binding of transcription activators^47–51^. The genomic arrangement of SRG1 and SER3 resembles that of *Chaserr* and *Chd2*, though the 3′ end of SRG1 overlaps SER3 promoter^48^, and the distance between their promoters is much shorter (~500 bp between SRG1 TSS and SER3 TSS vs. ~17 kb between the TSS of *Chaserr* and TSS of *Chd2*).

Another possibility is that the promoters of *Chaserr* and of *Chd2* compete for binding to shared enhancer elements, and elimination of the *Chaserr* promoter increases the ability of these enhancers to boost *Chd2* expression, as recently described for *MYC* and *PVT1*^52^. Both *Chaserr* and *Chd2* appear to be under the shared control of several enhancer regions found in the ~200-kb gene desert upstream of *Chaserr* (Fig. 2g and Supplementary Fig. 5), and those enhancers potentially regulate both genes together, which can explain the tight co-expression between *Chaserr* and *Chd2* (Fig. 1c). Indeed, deletion of the *Chaserr* promoter in mESCs leads to an increase in contacts between the *Chd2* promoter and three broad upstream enhancer regions (Fig. 2g). Enhancer competition may therefore contribute to the increase in *Chd2* expression in *Chaserr*^–/–^ cells. However, it is unlikely that such competition explains much of the CHD2 upregulation, as we observe similar levels of upregulation when using ASO Gapmers in different cellular systems (Fig. 2e, Fig. 3e, Supplementary Fig. 2b, c, and see below). In addition, removing just the gene body of *Chaserr*, which leaves the promoter intact, does not substantially affect Pol2 occupancy at the promoter (Supplementary Fig. 2a), nor the landscape of spatial contacts between the *Chd2* promoter and the upstream enhancers (Fig. 2g), despite leading to a similar upregulation of *Chd2* levels (Fig. 2e).

There is evidence that the RNA product of *Chaserr*, and not just its intact locus or the act of its production, are important for *Chd2* repression. The exon–intron architecture and sequence of *Chaserr* are highly conserved, suggesting that production of a particular RNA species, or splicing at particular locations, is important either because of the RNA product itself, or because they modulate the amount of transcription or Pol2 velocity upon encounter with the ~2-kb intergenic region between *Chaserr* and *Chd2*. The similar effects we observed when deleting *Chaserr* promoter or gene body, or when targeting the RNA with ASOs or Gapmers, also point to the importance of the RNA product, though we cannot rule out that ASOs and Gapmers may affect transcription by cleaving the nascent transcript.

Interestingly, in contrast to previous reports based on a gene trap inserted in intron 27 of *Chd2*^14,53,54^, nearly complete loss of CHD2 protein appears to be largely compatible with normal viability in mice in our hands, and in the similar mouse model generated by the EUCOMM consortium^15^. The previous observations of embryonic lethality following CHD2 loss of function can potentially be attributed to a dominant negative effect of the truncated protein product created by the gene trap. In contrast to the CHD2 loss models, we report here that increase in CHD2 levels brought about by loss of even a single copy of *Chaserr* is toxic and leads to perinatal lethality. We note that this is one of the most severe phenotypes reported so far for loss of a lncRNA^6,55^. The severe, CHD2-mediated phenotype is consistent with the high conservation of *Chaserr*–*Chd2* genomic organization and sequence during 500 million years of vertebrate evolution. Of the lncRNAs annotated in human and mouse, only ~5% (~100 genes) have evidence of conservation in fish^26,56^, and we were able to identify homologs of *Chaserr* in every vertebrate species we examined (Fig. 1a).

Our results have important implications from the therapeutic perspective. Individuals that bear mutations in the *CHD2* gene exhibit epilepsy and neurodevelopmental disorders^16^. In all described cases, these individuals are haploinsufficient for *CHD2*, and so bear an intact WT copy of CHD2. Therefore, increase of CHD2 expression through perturbation of *Chaserr*, e.g., by using antisense oligonucleotides, might have a therapeutic benefit. Importantly, *Chaserr* is highly conserved between human and mouse (Fig. 1a), and targeting of the human *CHASERR* by using Gapmers in human MCF7 and SH-SY5Y cells leads to an increase in *CHD2* mRNA and protein levels (Supplementary Fig. 6), suggesting that the results we observe in the mouse model are of direct relevance to human *CHASERR*.

Similarly to many other chromatin-remodeling factors that are increasingly implicated in human disease, the precise molecular function of *Chd2* and the direct consequences of its dysregulation are poorly understood. Here, we could leverage the similarity between the genomic arrangements of *Chaserr* and *Chd2* and those of the genes most affected by *Chaserr* perturbation to highlight the potential role of chromatin remodeling that CHD2 plays downstream of TTSs. Decoding lncRNA functions can therefore provide important insight into other layers of gene regulation. As chromatin modifiers are often flanked by lncRNAs, some as highly conserved as *Chaserr*, we expect that further research into the functions of these lncRNAs may uncover additional paradigms in chromatin biology.

## Methods

### Animals

The study was conducted in accordance with the guidelines of the Weizmann Institutional Animal Care and Use Committee (IACUC) and approved by it. C57black6 Ola HSD mice were purchased from Harlan Laboratories (Rehovot, Israel). All other mouse strains were bred and maintained at the Veterinary Resources Department of the Weizmann Institute.

### Generation of *Chaserr*^+/−^ mice

The *Chaserr*^**+/−**^ mice were generated as in Wang et al.^24^. All mice were generated by standard procedures at the Weizmann transgenic core facility. We used four single-guide RNAs (sgRNAs 1–4, see Supplementary Data 2), two targeting regions before the *Chaserr* transcription start site and two targeting regions in the first intron, which resulted in two founder lines. *Chaserr* mutant mice were genotyped by using primers flanking the targeted region followed by Sanger sequencing. To generate *Chd2* mutant mice we used one sgRNA targeted to the third exon of *Chd2* mRNA. *Chd2* mutant mice were genotyped by amplicon sequencing that identified a founder with a frameshift in the main ORF. Amplicon library preparation was as follows: *Chd2* mutant locus was amplified by using *Chd2* genotype nesting primers (Supplementary Data 2). Thereafter 1 µl of PCR reaction was used as template for the addition of R1 and R2 Illumina adaptors (R1/R2_Exon3_Chd2, Supplementary Data 2), followed by fragment AMPure (Beckman Coulter, A63881) size selection and cleanup. A second PCR reaction was used in order to add sequencing barcodes to the amplicons, followed by AMPure size selection. Indexed amplicons were pooled and sequenced on NextSeq 500. To genotype *Chd2*^*m*^ lines routinely, we used PCR with primers flanking the mutated region and digested the PCR product with the DdeI restriction enzyme whose recognition sequence is compromised in the mutated DNA. The fragment sizes were then analyzed on a 2% agarose gel. To generate *Chaserr*^*–*/–^ *Chd2*^m/m^ mice, we used *Chd2*^m/m^ background and continued as described above for generation of the *Chaserr*^*–*^ allele, by using only two sgRNAs (sgRNA2+3, Supplementary Data 2). sgRNA injection was done on CB6F1 Ola HSD mice that were later backcrossed with C57BL/6 Ola HSD. All the experiments were done on 4- to 15-week-old mice from F2 to F5 generations and E9.5–E18.5 developmental time points.

### Tissue culture

R1 mESCs (kind gift from the Nagy lab) were routinely cultured in mouse ES medium (mESM) consisting of 500 ml of DMEM (Gibco, 11965-092), 15% ES-grade Fetal Calf Serum (Biological Industries), sodium pyruvate 1 mM (Gibco, 11360-039), nonessential amino acids 1 × (Gibco, 11140-035), 0.1 mM b-mercaptoethanol (Sigma, M6250-250ML), penicillin–streptomycin (Biological Industries), and 1000 U/ml LIF (Weizmann Proteomics Unit).

All other cell lines (from ATCC) were routinely cultured in DMEM containing 10% fetal bovine serum and 100 U penicillin/0.1 mg ml^−1^ streptomycin at 37 °C in a humidified incubator with 5% CO_2_. Cell lines were routinely tested for mycoplasma contamination and were not authenticated.

Primary mEFs were isolated from E13.5 embryo. Heads were used to genotype the embryo, while the rest of the embryo (liver excluded) was dissociated with 1 mL of trypsin 0.05% in an Eppendorf tube for 5 min at 37 °C, pipetted for complete dissociation, and incubated for another 5 min at 37 °C. Cells were then supplemented with 2 mL of medium, centrifuged for 5 min at 192 × *g*. Cells were seeded on 10-cm gelatin-coated (0.1%) plates^57^.

### Neuronal differentiation

Neuronal differentiation was performed as previously described^58^. mESCs were first grown in the absence of mEFs for two passages and then seeded on gelatin-coated plates at a density of 1.5 × 10^5^ in N2B27 medium: 1:1 mixture of DMEM/F12 (Sigma) supplemented with N2 (Gibco), and Neurobasal medium (Gibco) supplemented with B27 (Gibco), 1× Glutamax (Gibco), 0.1 mM β-mercaptoethanol (Sigma), 100 U/ml penicillin, and 0.1 mg/ml streptomycin (Biological industries). After 4 days under these conditions, 3 × 10^5^ cells were replated on Poly-D-Lysine (Sigma, P6407) and Laminin (Life, 23017-015) coated plates, in N2B27 medium supplemented with 20 ng/ml FGF2 (Peprotech, 100-18B-50/100-18B-100). After 24 hr, FGF2 was removed and cells were cultured for 3 additional days^58^.

### Transfections

mESCs were transfected with electroporation of the Lonza protocol (http://bio.lonza.com/fileadmin/groups/marketing/Downloads/Protocols/Generated/Optimized_Protocol_309.pdf). HEK293T cells were transfected by using PolyEthylene Imine (PEI)^59^ (PEI linear, M*r* 25,000, Polyscience). Neuro2A transfection: 2 × 10^5^ cells were seeded in a six-well plate and transfected by using Lipofectamine 3000 (Life Technologies, L3000-008) with LNA1, LNA2, or a mix of LNA1–4 or with ASO1, ASO2, ASO3, or a mix of ASO1–3 to a final concentration of 50 nM. For transfection of MCF7 cells, 2 × 10^5^ cells were seeded in a six-well plate and transfected by using PEI with LNA h1 and/or LNA h2 to a final concentration of 50 nM. For transfection of SH-SY5Y cells, 2 × 10^5^ cells were seeded in a six-well plate and transfected by using DharmaFECT 4 Transfection Reagent (Dharmacon, T-2002-03) following the manufacturer’s protocol with LNA h1 and/or LNA h2 to a final concentration of 50 nM. Endpoints for all knockdown experiments were at 48 hr post transfection.

### Genome editing in mESCs

To generate *Chaserr*^p/p^ mESCs, 2 × 10^6^ mEF-depleted cells were transfected with sgRNAs 2 + 3 and pCas9_GFP (a gift from Kiran Musunuru, Addgene #44719). To generate *Chaserr*^b/b^ mESCs, 2 × 10^6^ cells were transfected with sgRNAs 2 + 5 and pCas9_GFP. The next day, fresh mESC medium was supplemented with 1 µg/ml puromycin (Invivogen, ant-pr-1) for 72 hr, while replacing medium every 24 hr. Next, 3–4 × 10^3^ cells were seeded at low density on a 10-cm plate until single colonies formed; thereafter, colonies were picked, expanded, genomic deletion was verified by PCR sequencing, and expression was tested by RT-qPCR and RNA-seq.

### RNA and RT-qPCR

Total RNA was extracted from different cell lines and mouse tissues, by using TRIREAGENT (MRC) according to the manufacturer’s protocol. cDNA was synthesized by using qScript Flex cDNA synthesis kit (95049, Quanta). Fast SYBR Green master mix (4385614) was used for qPCR.

### RNA-seq

Strand-specific mRNA-seq libraries were prepared from 500 to 4000 ng of total RNA by using the TruSeq Stranded mRNA (Illumina) or SENSE mRNA-Seq (Lexogen) library preparation kits, according to the manufacturer’s protocols. Libraries were sequenced on a NextSeq 500 to obtain 38–50-nt paired-end reads. Coverage tracks for the UCSC genome browser were prepared by aligning reads to the mm9 genome assembly with STAR^60^. Gene expression levels were quantified with RSEM^61^ and a RefSeq gene annotation database that was manually edited to correct the annotation of the last exon of *Chaserr*. Differential expression was computed with DESeq2 with default settings^62^. Genomic context was also analyzed by using the RefSeq gene annotations. RNA-seq and ATAC-seq datasets are deposited in GEO database under the accession GSE124375. RNA-seq data from previous studies were downloaded from the SRA database, and quantified with RSEM with the same annotation file.

### Western blot

Total protein was extracted from tissues and cell lines by lysis with RIPA supplemented with protease inhibitors and DTT 1 mM. Proteins were resolved on 8–10% SDS-PAGE gels and transferred to a polyvinylidene difluoride (PVDF) membrane. After blocking with 5% nonfat milk in PBS with 0.1% Tween-20 (PBST), the membranes were incubated with the primary antibody followed by the secondary antibody conjugated with horseradish peroxidase. Blots were quantified with Image Lab software. Primary antibodies were as follows: anti-Chd2 (Millipore, #MABE873, 1:1,000 dilution), anti-β-tubulin (Sigma, #T4026, 1:2,000 dilution). Secondary antibodies were as follows: anti-rat (#AP136P, 1:10,000 dilution), anti-mouse (#115-035-003, 1:10,000 dilution).

### ChIP-seq

In total, 1 × 10^7^ mEFs were cross-linked with formaldehyde at 1% final concentration, for 10 min at room temperature, and then quenched with 125 mM glycine for 5 min at room temperature. Cells were centrifuged for 5 min, 376 × *g* at 4 °C, and washed twice with PBS supplemented with PIC (protease inhibitor cocktail). Each pellet was then lysed in 1 mL of lysis buffer (5 mM PIPES, pH 8.0, 85 mM KCl, Igepal (10 µl/ml), and PIC), incubated for 15 min at 4 °C, and then centrifuged for 5 min at 21130 × *g* at 4 °C. Pellets were resuspended in 200 µl of nuclei lysis buffer (50 mM Tris-Cl, pH 8.1, 10 mM EDTA, 1% SDS, and PIC) for 30 min at 4 °C. Lysates were then sonicated (Bioruptor, #B01020001) for 12 cycles of 30 s ON, 30 s OFF. Pol2 antibody (5 µg, anti-Rbp1 NTD (D8L4Y), #14958) was bound to A/G magnetic beads in 1 mL of binding/washing buffer (PBS supplemented with 0.5% TWEEN and 0.5% BSA) for 1 hr at room temperature. Chromatin lysate was then diluted ×9 volume with IP dilution buffer (50 mM Tris-HCl, pH 7.4, 150 mM NaCl, 1% Igepal, 0.25% deoxycholic acid, and 1 mM EDTA, pH 8.0); the coupled beads were washed and added to the chromatin lysate and left for slow rotation overnight at 4 °C. Beads were handled as described^63^. Briefly, beads were washed five times with RIPA buffer (10 mM Tris-HCl, pH 8.0, 1 mM EDTA, pH 8.0, 140 mM NaCl, 1% Triton X-100, 0.1% SDS, and 0.1% Na-DOC), twice with RIPA-500 buffer (10 mM Tris-HCl, pH 8.0, 1 mM EDTA, pH 8.0, 500 mM NaCl, 1% Triton X-100, 0.1% SDS, and 0.1% Na-DOC), twice with LiCl buffer (10 mM Tris-HCl, pH 8.0, 1 mM EDTA, pH 8.0, 250 mM LiCl, 0.5% NP-40, and 0.5% Na-DOC), and once with TE buffer (10 mM Tris-HCl, pH 8.0 with 1 mM EDTA, pH 8.0). Beads were eluted with direct elution buffer (10 mM Tris-HCl, pH 8.0, 5 mM EDTA, pH 8.0, 300 mM NaCl, and 0.5% SDS) at room temperature. The eluate was incubated with RNaseA for 30 min at 37 °C, next with proteinase K for 2 hr at 37 °C, and last overnight at 65 °C. DNA was purified with the Agencourt AMPure XP system (Beckman Coulter Genomics, A63881). Libraries were constructed as previously described^63^ and sequenced with paired-end sequencing on Illumina NextSeq 500.

### ATAC-seq

ATAC-seq was performed as previously described^32^. We isolated nuclei from 25 × 10^3^ mEFs derived from WT, *Chaserr*^–/–^, or *Chaserr*^–/–^ *Chd2*^m/m^ backgrounds. Cells were centrifuged at 500 g for 5 min, followed by a wash with 50 μl of cold 1x PBS and centrifugation at 500 × *g* for 5 min. Cells were lysed with cold lysis buffer (10 mM Tris-Cl, pH 7.4, 10 mM NaCl, 3 mM MgCl_2_, and 0.1% IGEPAL CA-630). Immediately after lysis, nuclei were spun at 500×g for 10 min at 4 °C. Immediately following the nuclei prep, the pellet was resuspended in the transposase reaction mix (12.5 μL of 2× TD buffer, 1 μL of Transposase (Illumina), and 11.5 μL of nuclease-free water). The transposition reaction was carried out for 1 hr at 37 °C. Directly following transposition, the reaction was treated with 2 μL of 5% SDS, 2 μL of 20 mg/ml proteinase K, and 5 μl of cleanup buffer (900 mM MaCl, 30 mM EDTA) for 30 min at 40 °C. The sample was purified by using 2× SPRI beads. Following purification, we amplified library fragments with 2× Kappa HiFi and 1.25 μM of i5 and i7 primers (Illumina) using the following PCR conditions: 98 °C for 2 min, 98 °C for 20 s, followed by thermocycling at 63 °C for 30 s and 72 °C for 1 min. We amplified the libraries for nine cycles. The libraries were purified by using 0.5× and 1.8× for double size selection cut off. Next we amplified the libraries by using a second PCR with the same conditions for five cycles. Lastly, the libraries were purified by using 2× SPRI beads. Libraries were sequenced with paired-end sequencing on Illumina NextSeq 500.

### ATAC-seq and ChIP-seq data analysis

Reads were aligned to the mm9 genome assembly with Bowtie2^64^, and ATAC-seq peaks were called by MACS2^65^. Normalized read coverage files were computed by MACS2. ATAC-seq peaks from individual samples were merged with bedtools^66^. Read coverage was quantified by using Homer 4.9.1^67^. Differential accessibility was computed with DESeq2^62^ called by Homer^67^ using an annotation file based on RefSeq gene models, considering only genes longer than 2 kb. Each gene was assigned with a single TSS based on FANTOM5 CAGE annotations for mEFs/mESCs, and with the most distal annotated TTS. The promoter region for each gene was defined as ±300 around that TSS, gene body as the region from 1 kb downstream of the TSS to the TTS, and the region downstream of the TTS as 2 kb from the TTS. ENCODE Pol2 ChIP-seq data (ENCFF001LOL accession) and CHD2 MNaseq-seq data were processed the same way. Pol2 pausing index was computed as the ratio between the Homer-normalized average Pol2 occupancy in the promoter and the gene body (after adding a pseudocount of 0.5 to the total coverage in each region). Only genes with at least five normalized reads per kb in the gene body were considered.

### Targeted chromosome conformation capture (4C)

Following depletion from mEFs by 20-min incubation on gelatin-coated plates, 3C was carried out on 5 × 10^6^ mESCs essentially as described^68^, with the following slight modification: permeabilization buffer constitution was 10 mM Tris-HCl, pH 8, 10 mM NaCl, and 0.5% NP-40, supplemented with protease inhibitors. 4C libraries were prepared as described^69^, with primers directed to the promoter region of *Chd2* (upstream primer sequence: GCTCAAGCACCCTTTTTAAGCCAG, downstream primer sequence: AATGATACGGCGACCACCGAGATCTACACTCTTTCCCTACACGACGCTCTTCCGATCTGAAATGTAATTTGTTCCTTTTGTC). Libraries were sequenced on Illumina NextSeq 500, and analyzed as described^69^.

### Single-molecule FISH

Stellaris probe libraries targeting *Chaserr* introns (48 probes) or exons (19 probes) (Supplementary Data 3) were designed by using the Biosearch Technologies server and ordered from Biosearch Technologies. Single-molecule FISH was done as described^70,71^. Briefly, mEFs were plated on poly-l-lysine and 0.1% gelatin-coated coverslips. Hybridizations (25% formamide) were done overnight with CAL Fluor^®^ Red 590 Dye (*Chaserr* introns) and Quasar 670 (*Chaserr* exons) fluorophores. Nuclear staining was performed with DAPI (Sigma-Aldrich, D9542). iXon Ultra 888 EMCCD camera and Nikon’s NIS-Elements were used for imaging.

### Cycloheximide treatment

mESCs and mEFs were treated with DMSO (vehicle) or CHX (Sigma #C7698) 100 µg/mL and collected at the indicated time points for RNA/protein analysis.

### Extraction of cytoplasmic and nuclear RNA

Cells were washed twice with ice-cold PBS, then scraped with ice-cold buffer A (EGTA 15 µM, EDTA 10 µM, protease inhibitor cocktail (Sigma, P8340), and RNase inhibitor (ERX-E4210-01)), and centrifuged at 400 × *g*, 4 °C for 5 min. The supernatant was discarded, fresh buffer A was added, and the pellet was mechanically pipetted with 21G followed by a 27G syringe. Cells were then centrifuged at 2000 × *g*, 4 °C for 5 min and the syringe step was repeated. The cells were then centrifuged at 2500 × *g*, 4 °C for 5 min. The pellet was then kept as the nuclear fraction, and the supernatant was centrifuged again at 6000 × *g*, 5 min followed by another supernatant collection (clean cytosolic fraction). Nuclear pellet was then washed three times with buffer A. RNA was extracted with TRIREAGENT (MRC).

### CRISPR guide RNA cloning

Guide RNAs were designed by CHOPCHOP^72^. Cloning of plasmids was done following Zhang Lab General Protocol (http://www.genome-engineering.org/crispr/wp-content/uploads/2014/05/CRISPR-Reagent-Description-Rev20140509.pdf) by using phU6-gRNA^73^ (a gift from Charles Gersbach, Addgene plasmid #53188) or pKLV-U6gRNA(BbsI)-PGKpuro2ABFP^74^ (a gift from Kosuke Yusa, Addgene plasmid #50946).

### *Chaserr* CRISPR activation

In total, 2 × 10^5^ Neuro2a cells were seeded in six-well plates and transfected with dCas9-VP64 (Addgene #61422, at 1 µg) and five gRNAs (#3,4,8–10, Supplementary Data 2) designed to target a region upstream of the *Chaserr* promoter (100 ng in total). As a control, an empty gRNA vector was used. Cells were harvested 48 hr later and TRIZOL was used for RNA isolation.

### *Chaserr* cloning and lentiviral production

cDNA from Source BioScience clone C130076G01 was amplified with PCR adding restriction sites for NheI at 5′ end and AgeI at 3′ end (Supplementary Data 2) and cloned into pLIX_402 vector (a gift from David Root, Addgene #41394) by using restriction ligation. To produce viruses, HEK293T (2.5 × 10^6^, 10-cm plate) cells were transfected by using PEI with psPAX2 (3.5 µg, a gift from Didier Trono, Addgene #12260), pMD2.G (1.5 µg, a gift from Didier Trono, Addgene, #12259), and pLIX_402-Chaserr (5 µg). Viruses were collected 48 hr post transfection and filtered through 0.45-µm sterile filters. Viruses were supplemented with polybrene (1:1000, Sigma, #107689-10 G) upon cell infection.

### 3′ RACE

3′ RACE was performed with RNA from WT mEFs by using SMARTer RACE 5′/3′ kit (Clontech, #634859). Briefly, RACE products were amplified by using nested primers CCCCGCTTGAAGAGTTTGAAATGGAC and GATTACGCCAAGCTT TACCACTGAGAAATCAAGATGGCAG. Amplification resulted in a single PCR band that was then purified from 1% agarose gel by using NucleoSpin (#740609.50), cloned into the pRACE vector (provided with the kit), and transformed into Stellar competent cells. RACE products were Sanger sequenced with M13F primer: TGTAAAACGACGGCCAGT and aligned to the mouse genome.

### Micro-CT scanning and analysis

Prior to micro-CT scanning, the mice were anesthetized by using IP injection of a mix of xylazine (10 mg/kg) and ketamine (100 mg/kg). Mice were scanned by using a micro-CT device TomoScope^®^ 30 S Duo scanner equipped with two source–detector systems. The scanner uses two X-ray sources and a detector system that are mounted on a gantry that rotates around a bed holding the animal. The operation voltages of both tubes were 40 kV. The integration time of protocols was 90 ms (360 rotations) for 3-cm length, and axial images were obtained at an isotropic resolution of 80 μm. Due to the maximum length limit, to cover the whole mouse body, imaging was performed in two–three parts with the overlapping area, and then all slices merged to one dataset representing the entire ROI. The radiation dose range was 0.9 Gy. All micro-CT scans were reconstructed by using a filtered back-projection algorithm using scanner software. Then the reconstructed datasets for each mouse were merged to one dataset by using ImageJ software. 3D volume rendering images were produced by using Amira Software.

### RNA immunoprecipitation (RIP)

Neuro2a cells (ATCC) were collected, centrifuged at 94 × *g* for 5 min at 4 °C, and washed twice with ice-cold phosphate-buffered saline (PBS) supplemented with ribonuclease inhibitor (100 U/mL, #E4210-01) and protease inhibitor cocktail (Sigma-Aldrich, #P8340). Next, cells were lysed in 1 mL of lysis buffer (5 mM PIPES, 200 mM KCl, 1 mM CaCl_2_, 1.5 mM MgCl_2_, 5% sucrose, 0.5% NP-40, supplemented with protease inhibitor cocktail + 100 U/ml RNase inhibitor, and 1 mM DTT) for 10 min on ice. Lysates were sonicated (Vibra-cell VCX-130) three times for 1 s ON, 30 s OFF at 30% amplitude. Chilled tube holders were used and swapped between shearing runs to reduce temperature elevation. Lysates were centrifuged at 21130 × *g* for 10 min at 4 °C. Supernatants were then transferred to new 2-mL tubes and supplemented with 1 mL of IP binding/washing buffer (150 mM KCl, 25 mM Tris (pH 7.5), 5 mM EDTA, 0.5% NP-40, supplemented with protease inhibitor cocktail + 100  U/ml RNase inhibitor, and 0.25 mM DTT). The samples were then rotated for 2–4 hr at 4 °C with 5 µg of antibody per reaction. Meanwhile, 50 µl of beads (Dynabeads sheep anti-rat, #11035, or GenScript A/G beads #L00277) per reaction were washed three times with IP binding/washing buffer, followed by addition to lysates for an overnight rotating incubation. On the next day, the beads were washed three times in IP binding/washing buffer and resuspended in 0.5 mL of TRIZOL for RNA extraction. To generate sequencing libraries, we first depleted ribosomal RNA with Lexogen RiboCop rRNA depletion kit V1.2 (#037), followed by Lexogen SENSE total RNA-seq library prep kit (#042). Libraries were sequenced on NextSeq 500 by using paired-end 40-nt reads. Reads were mapped to the mouse mm9 genome assembly by using STAR. Exonic and intronic reads were tallied by using Picard CollectRnaSeqMetrics tool. Reads were then counted by using Homer and an annotation file constructed as described above for Pol2 ChIP-seq analysis, but containing the whole gene body of the selected isoform (from TSS to TTS). Differential expression was evaluated by using DESeq2 as implemented in Homer (as for the Pol2 ChIP-seq data).

### Reporting summary

Further information on research design is available in the Nature Research Reporting Summary linked to this article.

## Supplementary information

Supplementary Information

Peer Review

Reporting Summary

Description of Additional Supplementary Files

Supplementary Data 1

Supplementary Data 2

Supplementary Data 3

Source Data

## Data Availability

A reporting summary for this article is available as a Supplementary Information file. RNA-seq, ATAC-seq, ChIP-seq, RIP-seq, and 4C datasets are deposited in GEO database under the accession GSE124375. The source data underlying Figs. 1b–c, e–f, 2a–c, f–g, 3c–j, 4a–g, 5a, c, f, and 6a–f are provided as Source Data file. All data are available from the corresponding author upon reasonable request.
