# Supplementary material for: Regulation of CHD2 expression by the Chaserr long noncoding RNA gene is essential for viability
Source: Nat Commun. 2019 Nov 8;10:5092. doi: 10.1038/s41467-019-13075-8 (PMC6841665; doi:10.1038/s41467-019-13075-8)
Supplement: Supplementary file 1 — Supplementary Information [file 41467_2019_13075_MOESM1_ESM.pdf]

Supplementary Information for "Regulation of CHD2 expression by the Chaserr long noncoding RNA gene is essential for viability" by Rom et al.

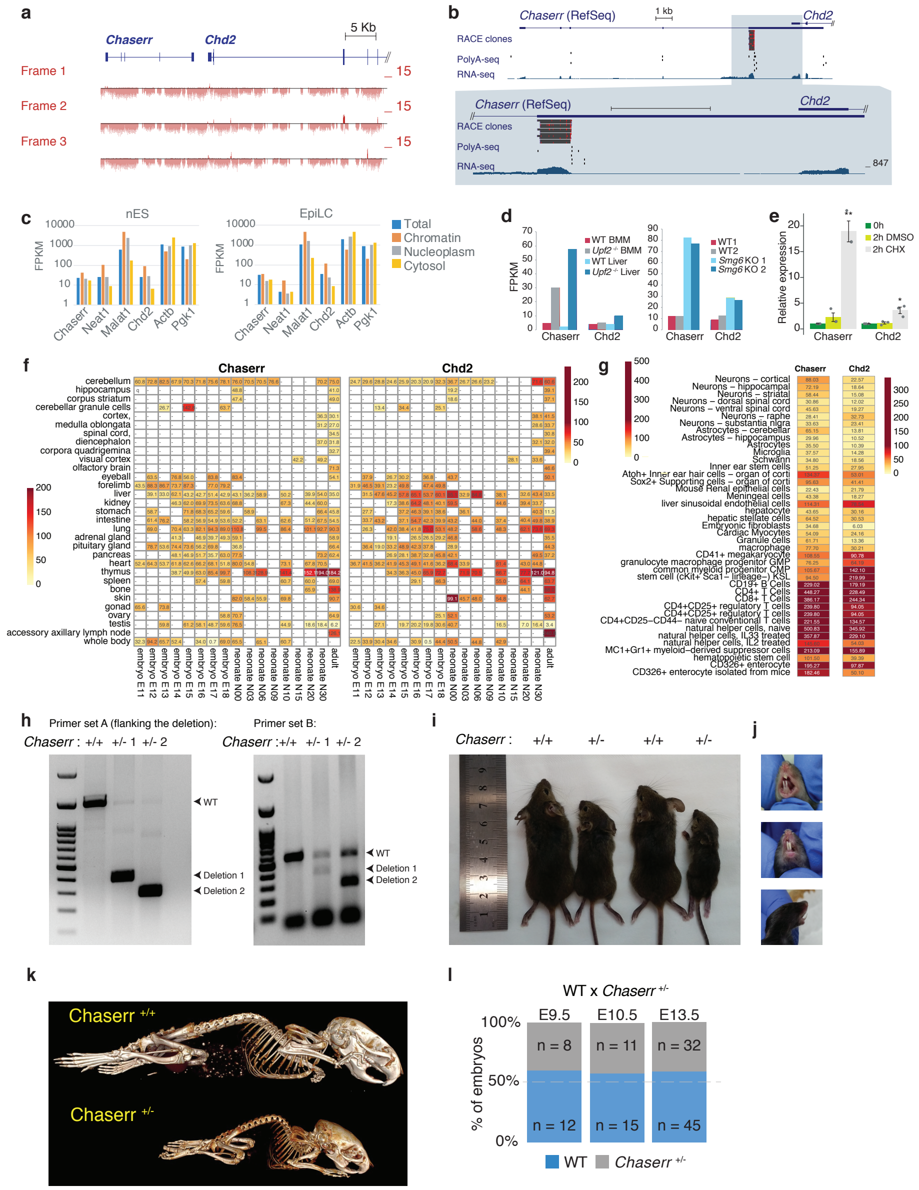

Supplementary Figure 1 | *Chaserr* haploinsufficiency leads to pleiotropic phenotype in mice. a, PhyloCSF scores[1] throughout the *Chaserr* locus in the three possible frames. **b,** 3' RACE on RNA extracted from mEFs. Shown are BLAT alignments of Sanger sequencing of ten independent clones, alongside mEF RNA-seq and PolyA-seq clusters from[2]. **c,** Expression levels of the indicated RNAs in the indicated compartment of naïve embryonic stem cells (nES) or epiblast-like cells (EpiLCs) (taken from GEO GSE99366). **d,** RNA-seq-based levels of gene expression in WT and *Upf2*$^{-/-}$ bone marrow macrophages (BMM) and liver (left), taken from[3] and in WT and Smg6$^{-/-}$ mESCs (right), taken from[4]. **e,** Changes in gene expression following treatment of mESCs with cyclohexamide (CHX) or DMSO (vehicle control). Normalized to *Actb*. n=3. **f-g,** *Chaserr* and *Chd2* expression in FANTOM5 CAGE data in tissues from the indicated developmental and neonatal stages (d) and cell types (e). **h,** Detection of the genomic deletion of *Chaserr* promoter and first exon using PCR with flanking primers (set A) or with three primers (set B) that differentiate between *Chaserr*$^{+/-}$ and *Chaserr*$^{-/-}$ genotypes. **i,** *Chaserr*$^{+/+}$ and *Chaserr*$^{+/-}$ four-week old females (left) and males (right). **j,** Representative images of malocclusion observed in *Chaserr*$^{+/-}$ mice. **k,** Representative computed tomography (CT) scan image of *Chaserr*$^{+/+}$ and *Chaserr*$^{+/-}$ six weeks old mice. **l,** Genotypes of embryos from a cross of WT and *Chaserr*$^{+/-}$ mice, at the indicated ages. Error bars show S.E.M. *P<0.05, **P<0.01 (two-sided t-test). Source data are provided as a Source Data file.

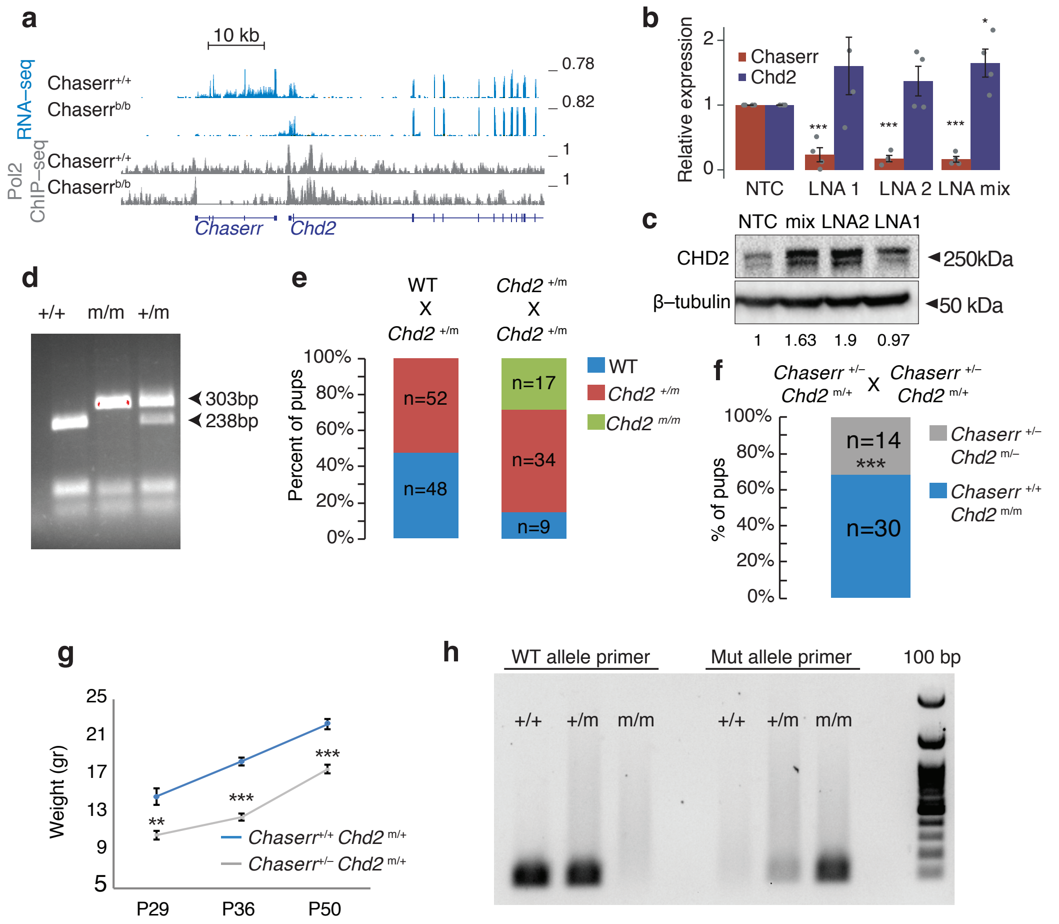

Supplementary Figure 2 | Additional characterization of *Chaserr* loss of function models. a, RNA-seq and Pol2 ChIP-seq read coverage in the indicated mESC lines. **b,** qRT-PCR of the indicated genes in Neuro2a cells following transfection of the indicated LNA Gapmers. Normalized to *Actb*. n=4. **c,** Western blots for the same samples as in a. **d,** Genotyping of *Chd2*^{m} allele using PCR followed by DdeI digestion. Expected size of the WT (cut) band is 238 and of the mutated (uncut) band is 303 bp. **e-f,** Survival rates of pups from the indicated crosses. **g,** Weight in grams of male pups from crosses of *Chaserr*$^{+/-}$ and *Chd2*$^{m/m}$ mice. n=4–10 mice for each genotype per time point. **h,** PCR analysis of mEFs with the indicated genotypes using the indicated primer sets for distinguishing between *Chd2*$^{+}$ and *Chd2*^{m} alleles. Error bars show S.E.M. *P<0.05 **P<0.01 and ***P<0.001 (two-sided t-test). Source data are provided as a Source Data file.

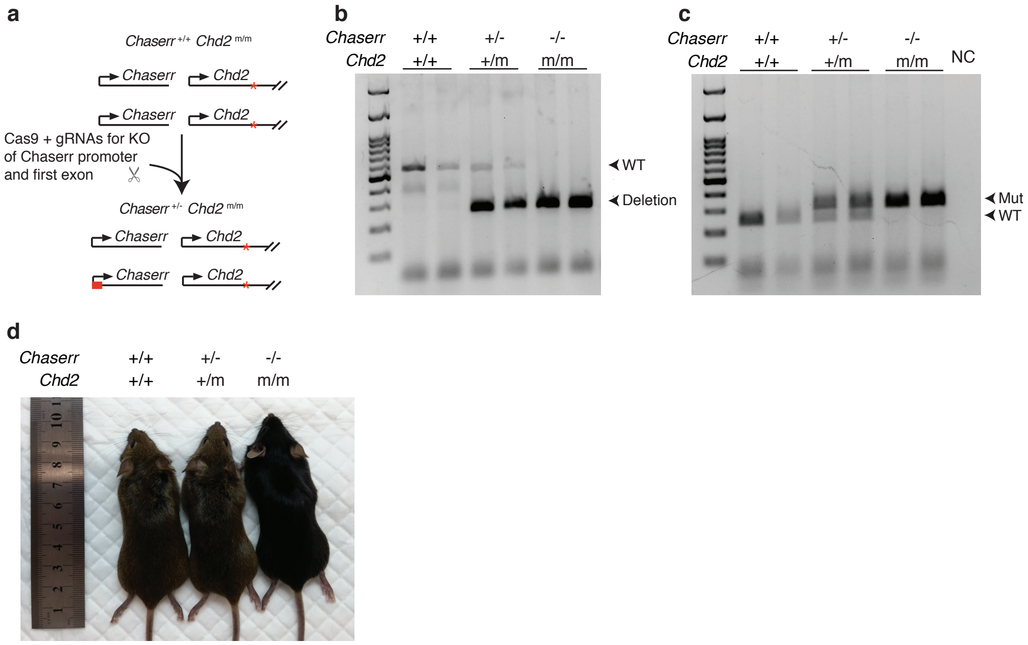

Supplementary Figure 3 | Rescue of loss of *Chaserr* by a hypomorphic allele of *Chd2*. a, Scheme of the generation of *Chaserr*$^{+/-}$ *Chd2*$^{+/m}$ transgenic mice. **b,** PCR with primer set B (**Supplementary Fig. 1h**) for identifying mice with the indicated genotypes. **c,** Genotyping of *Chd2*^{m} alleles using PCR followed by DdeI digestion. Expected size of the WT (cut) band is 238 and of the mutated (uncut) band is 303 bp. **d,** Representative mice with the indicated genotypes.

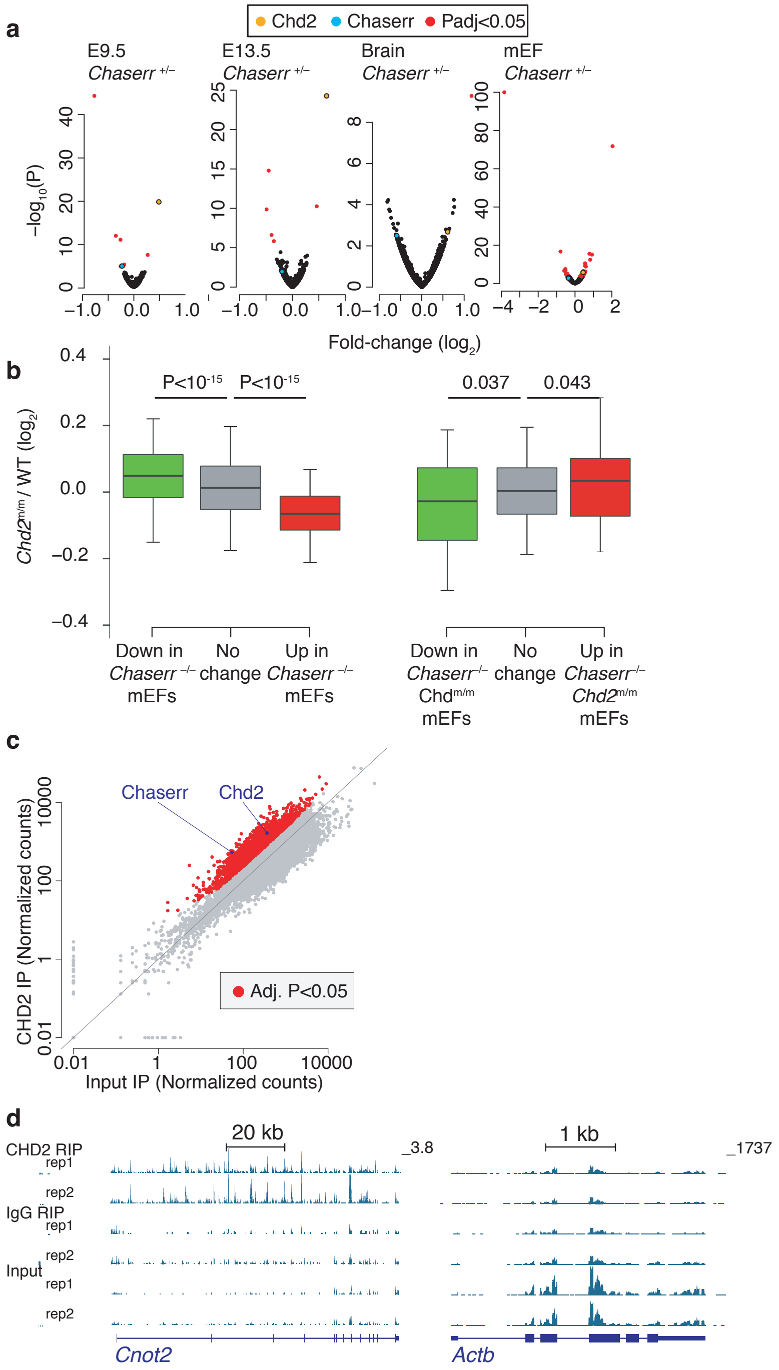

Supplementary Figure 4 | RNA-seq and RIP-seq analyses. a, Changes in gene expression in the indicated *Chaserr*$^{+/-}$ samples relative to WT controls. Fold-changes and P-values computed using DESeq2. **b,** Changes in gene expression (computed using DESeq) in *Chd2*$^{m/m}$ mEFs relative to WT controls for genes significantly (>25% fold-change and adjusted P<0.05) differentially expressed in mEFs from the indicated background relative to WT controls. P-values computed using Wilcoxon rank sum test. Boxplots show the 5th, 25th, 50th, 75th and 95th percentiles. **c,** Normalized RIP-seq read counts within gene bodies in CHD2 RIP and Input libraries (average of two replicates). Red dots correspond to genes with adjusted P<0.05 (DESeq2 analysis). **d,** RIP-seq read coverage in the *Cnot2* locus (significantly enriched in CHD2 RIP libraries compared to IgG and input controls) and in *Actb* locus (negative control, not enriched). Source data are provided as a Source Data file.

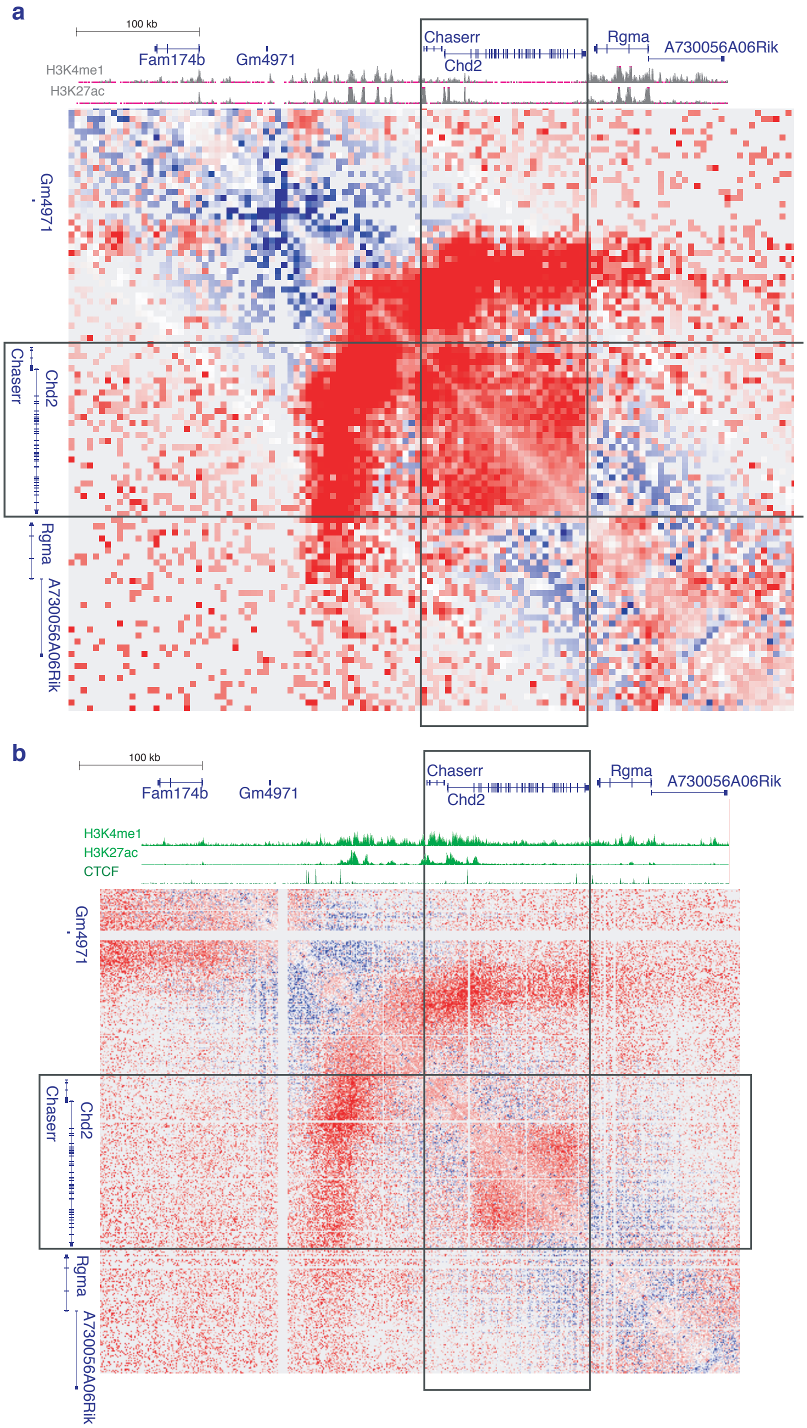

Supplementary Figure 5 | Spatial contacts in the *Chaserr*/*Chd2* locus. Hi-C data from adult mouse brain[5] (**a**) and mESCs[6] (**b**) visualized using JuiceBox[7]. Regions with excess of contacts over expected are in red and those with depletion of contacts are in blue. The box surrounds the *Chaserr*/*Chd2* locus. H3K4me1 and H3K27ac chromatin marks and CTCF ChIP-seq tracks are from the ENCODE project data for mouse whole brain (a) and mESCs (b).

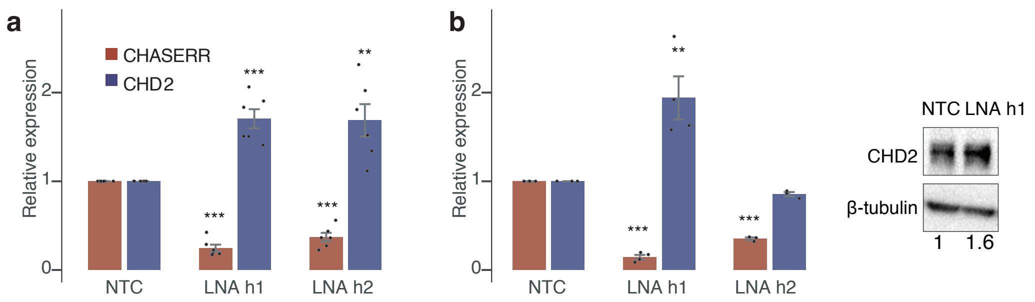

Supplementary Figure 6 | Knockdown of *CHASERR* leads to increase in CHD2 in human cells. a, qRT-PCR of the indicated RNA following transfection of the LNA Gapmers targeting *CHASERR* or non-targeting control (NTC) in MCF-7 cells. Normalized to *Actb*. n=6. **b,** Left: As in a, for SH-SY5Y cells, n=4. Right: A representative western blot CHD2 in cells transfected with LNA h1 Gapmer for *CHASERR* or a non-targeting control. Error bars show S.E.M. **P<0.01 and ***P<0.001 (two-sided t-test). Source data are provided as a Source Data file.

Supplementary References

1. Lin, M. F., Jungreis, I. & Kellis, M. PhyloCSF: a comparative genomics method to distinguish protein coding and non-coding regions. *Bioinformatics* **27**, i275–82 (2011).
2. Derti, A. *et al.* A quantitative atlas of polyadenylation in five mammals. *Genome Res.* **22**, 1173–1183 (2012).
3. Weischenfeldt, J. *et al.* Mammalian tissues defective in nonsense-mediated mRNA decay display highly aberrant splicing patterns. *Genome Biol.* **13**, R35 (2012).
4. Li, T. *et al.* Smg6/Est1 licenses embryonic stem cell differentiation via nonsense-mediated mRNA decay. *EMBO J.* **34**, 1630–1647 (2015).
5. Deng, X. *et al.* Bipartite structure of the inactive mouse X chromosome. *Genome Biol.* **16**, 152 (2015).
6. Bonev, B. *et al.* Multiscale 3D Genome Rewiring during Mouse Neural Development. *Cell* **171**, 557–572.e24 (2017).
7. Durand, N. C. *et al.* Juicebox Provides a Visualization System for Hi-C Contact Maps with Unlimited Zoom. *Cell Syst* **3**, 99–101 (2016).
