# Supplementary material for: Regulation of CHD2 expression by the Chaserr long noncoding RNA gene is essential for viability
Source: Nat Commun. 2019 Nov 8;10:5092. doi: 10.1038/s41467-019-13075-8 (PMC6841665; doi:10.1038/s41467-019-13075-8)
Supplement: Supplementary file 3 — Reporting Summary [file 41467_2019_13075_MOESM3_ESM.pdf]

nature research

Corresponding author(s): *Double-blind peer review submissions: write DBPR and your manuscript number here instead of author names.*

Last updated by author(s): *YYYY-MM-DD*

Reporting Summary

Nature Research wishes to improve the reproducibility of the work that we publish. This form provides structure for consistency and transparency in reporting. For further information on Nature Research policies, see Authors & Referees and the Editorial Policy Checklist .

Statistics

For all statistical analyses, confirm that the following items are present in the figure legend, table legend, main text, or Methods section.

n/a	Confirmed	
☐	☒	The exact sample size (*n*) for each experimental group/condition, given as a discrete number and unit of measurement
☐	☒	A statement on whether measurements were taken from distinct samples or whether the same sample was measured repeatedly
☐	☒	The statistical test(s) used AND whether they are one- or two-sided<br>*Only common tests should be described solely by name; describe more complex techniques in the Methods section.*
☒	☐	A description of all covariates tested
☐	☒	A description of any assumptions or corrections, such as tests of normality and adjustment for multiple comparisons
☒	☐	A full description of the statistical parameters including central tendency (e.g. means) or other basic estimates (e.g. regression coefficient) AND variation (e.g. standard deviation) or associated estimates of uncertainty (e.g. confidence intervals)
☐	☒	For null hypothesis testing, the test statistic (e.g. *F*, *t*, *r*) with confidence intervals, effect sizes, degrees of freedom and *P* value noted<br>*Give P values as exact values whenever suitable.*
☒	☐	For Bayesian analysis, information on the choice of priors and Markov chain Monte Carlo settings
☒	☐	For hierarchical and complex designs, identification of the appropriate level for tests and full reporting of outcomes
☐	☒	Estimates of effect sizes (e.g. Cohen's *d*, Pearson's *r*), indicating how they were calculated

Our web collection on statistics for biologists contains articles on many of the points above.

Software and code

Policy information about availability of computer code

Data collection	bcl2fastq 2.20.0.422
Data analysis	Bowtie 2.2.9; STAR 2.5.3a; RSEM 1.3.1; R 3.3.2; ngsplot 2.61; Homer 4.9.1

For manuscripts utilizing custom algorithms or software that are central to the research but not yet described in published literature, software must be made available to editors/reviewers. We strongly encourage code deposition in a community repository (e.g. GitHub). See the Nature Research guidelines for submitting code & software for further information.

Data

Policy information about availability of data

All manuscripts must include a data availability statement. This statement should provide the following information, where applicable:

- Accession codes, unique identifiers, or web links for publicly available datasets
- A list of figures that have associated raw data
- A description of any restrictions on data availability

GEO GSE124375

Field-specific reporting

Please select the one below that is the best fit for your research. If you are not sure, read the appropriate sections before making your selection.

☒ Life sciences ☐ Behavioural & social sciences ☐ Ecological, evolutionary & environmental sciences

For a reference copy of the document with all sections, see nature.com/documents/nr-reporting-summary-flat.pdf

Life sciences study design

All studies must disclose on these points even when the disclosure is negative.

Sample size	No specific methods were used for sample size estimation
Data exclusions	No data were excluded
Replication	All conclusions were replicated
Randomization	No randomization was used
Blinding	No blinding was used

Reporting for specific materials, systems and methods

We require information from authors about some types of materials, experimental systems and methods used in many studies. Here, indicate whether each material, system or method listed is relevant to your study. If you are not sure if a list item applies to your research, read the appropriate section before selecting a response.

Materials & experimental systems

n/a	Involved in the study	
☐	☒	Antibodies
☐	☒	Eukaryotic cell lines
☒	☐	Palaeontology
☐	☒	Animals and other organisms
☒	☐	Human research participants
☒	☐	Clinical data

Methods

n/a	Involved in the study	
☐	☒	ChIP-seq
☒	☐	Flow cytometry
☒	☐	MRI-based neuroimaging

Antibodies

Antibodies used	anti-pol2, Cell Signaling, 14958, Rbp1 NTD (D8L4Y) | anti-Chd2, Millipore, MABE873, clone 8H3, lot: 2991839 | anti-beta tubulin, Sigma, T4026, clone TUB 2.1, lot: 043M4785
Validation	For anti-pol2, which was used for ChIP, from distributor website: For optimal ChIP and ChIP-seq results, use 10 µl of antibody and 10 µg of chromatin (approximately 4 x 106 cells) per IP. This antibody has been validated using SimpleChIP® Enzymatic Chromatin IP Kits.<br><br>For anti-Chd2: From distributor website: "This Anti-Chd2 antibody, clone 8H3 is validated for use in western blotting"<br><br>For anti-beta tubulin: From distributor website: antibody has been validated for western blot

Eukaryotic cell lines

Policy information about cell lines

Cell line source(s)	R1 mESCs from Nagy lab, all other lines from ATCC
Authentication	Cell lines used were not authenticated
Mycoplasma contamination	We confirm that all cells were tested for mycoplasma, and found negative
Commonly misidentified lines<br>(See ICLAC register)	No commonly misidentified cell lines were used

Animals and other organisms

Policy information about studies involving animals; ARRIVE guidelines recommended for reporting animal research

Laboratory animals	Mice founders were generated using CB6F1 and backcrossed with C57BL/6. Study was conducted and males and females interchangeably from E9.5 to 16 week old mice.

Wild animals	This study did not involve wild animals
Field-collected samples	N/A
Ethics oversight	Weizmann Institutional Animal Care and Use Committee (IACUC)

Note that full information on the approval of the study protocol must also be provided in the manuscript.

ChIP-seq

Data deposition

- [x] Confirm that both raw and final processed data have been deposited in a public database such as GEO.
- [x] Confirm that you have deposited or provided access to graph files (e.g. BED files) for the called peaks.

Data access links<br>*May remain private before publication.*	GEO GSE124375
Files in database submission	bigWigCoverage files and raw FASTQ datano peak calling was performed
Genome browser session<br>(e.g. UCSC)	bigWig tracks are available in the GEO submission

Methodology

Replicates	2 replicates for each condition in mEFs
Sequencing depth	At least 10 million reads per replicate
Antibodies	anti-pol2, Cell Signaling, 14958, Rbp1 NTD (D8L4Y)
Peak calling parameters	No peak calling was used for ChIP-seq data
Data quality	bigWig visual inspection
Software	Bowtie2 for read mapping, Homer for read counting
