# Supplementary material for: Regulation of CHD2 expression by the Chaserr long noncoding RNA gene is essential for viability
Source: Nat Commun. 2019 Nov 8;10:5092. doi: 10.1038/s41467-019-13075-8 (PMC6841665; doi:10.1038/s41467-019-13075-8)
Supplement: Supplementary file 4 — Description of Additional Supplementary Files [file 41467_2019_13075_MOESM4_ESM.docx]

Description of Additional Supplementary Files

File Name: Supplementary Data 1
Description: Mouse Phenotype and Gene Ontology enrichments for genes up- and down-regulated in Chaserr–/– mEFs.

File Name: Supplementary Data 2
Description: Sequences of primers, ASOs, and GapmeRs used in this study.

File Name: Supplementary Data 3
Description: Stellaris smFISH probe sequences.
