# Supplementary material for: Regulation of CHD2 expression by the Chaserr long noncoding RNA gene is essential for viability
Source: Nat Commun. 2019 Nov 8;10:5092. doi: 10.1038/s41467-019-13075-8 (PMC6841665; doi:10.1038/s41467-019-13075-8)
Supplement: Supplementary file 2 — Peer Review [file 41467_2019_13075_MOESM2_ESM.pdf]

Reviewers' comments:

Reviewer #1 (Remarks to the Author):

The study by Rom and colleagues analyses the conserved lncRNA locus Chaserr, upstream of the Chd2 gene, which encodes a chromatin modifying protein essential for development and the human orthologue is implicated in human pathogenesis. The authors show that Chaserr's only function is to negatively regulate Chd2 expression levels. The authors characterize the effect from excess amount of Chd2 protein, but do not provide anything how Chaserr regulates Chd2; which I think is the burning question. Although Chaserr is an interesting locus, this work combines data from sometimes random samples and tissues. Why do the authors conclude that just because Chd2 is active in most tissue one cannot stick to one particular tissue/cell type for analysis? It is well established that pleiotropic expressed genes still have distinct functions in specific cell types.
It remains completely unclear if the RNA is the carrier of the function or the transcriptional process; I see the latter to be major part. An interesting part of this work is that blocking the RNA of Chaserr could be of therapeutic potential to adjust Chd2 function in patients that have a functional inactive Chd2 allele. In that direction the RNA vs locus issue must be resolved.
If the following points are not addressed comprehensively, I cannot recommend publication in Nature Communications.

Major points:
1) The authors claim that Chaserr RNA is localized, or at least enriched, in the nucleus. From their data in Fig 1b this is hardly visible. Why do the authors analyze mouse duodenum, a tissue known to have a high autofluorescence activity? The data are not convincing and multiple spots in the nuclei are seen in Fig 1b. They isolated MEFs later in their study and the RNA FISH data must be repeated in an ex vivo cell type. Some convincing RNA FISH pictures are seen in Cabili, Genome Bology, 2016. In addition, the RNA FISH have to be correctly controlled by performing them in parallel in Chaserr null MEFs. These data must be complemented with a fractionation analysis of chromatin/nucleus/cytosol and not solely nucleus and cytosol. Again, it is important to refine this part for the subsequent points in this study -> while overexpression of Chaserr has no effect on Chd2 expression, LNA based Knock-down has an effect. It must be clearly shown that Chaserr RNA resides at is locus and eventually at its origin of production.
2) Following the previous point: If exogenous overexpression of Chaserr has no effect, CRISPR-i guided overexpression of the endogenous locus can be performed. This would also clarify the point of RNA production at its endogenous locus is regulating Chd2. If the authors tried this without success: either show the data or try it in a different cell type than ESCs. A minimum of two different cell types must be tested before one can conclude that CRISPR-i overexpression doesn't work on this locus.
3) In the initial Chaserr mutant analysis the author show that they never find homozygous pubs. At what stage are the embryos showing first defects? The authors have to determine at least the timepoint when the Chaserr homozygous embryos are dying. Later the show that homozygous ESCs are viable, so some development should occur. See more below point 5.
4) The cis-acting activity in the heterozygous compound mutant Chaserr/Chd2 hints towards a Chaserr RNA independent function. Therefore, the lncRNA locus rather than the RNA itself is active. In that direction the title is misleading and must be changed: the lncRNA locus regulates Chd2 not the RNA. I see strong similarities with the PVT1 locus work (Cho at al., Cell, 2018) from the Chang lab, where they show a function of the lncRNA PVT1 promotor on the cis gene expression. The authors provide ExtFig4 (why adult mouse brain?) that only shows two major domains: 1) the super-enhancer region upstream of Chaserr and the tight interaction of the Chaserr/Chd2 region. Eventually, Chaserr is the 'border' and by deleting Chd2 promoter (again: see PVT1) they collapsed this separation and 3C analysis could show this.
5) The RNA-seq analysis in Fig 4 is confusing and not straight forward; the authors compare expression profiles of random tissues, cells and structures. Why do the authors only compare het embryos and not homozygous? Please explain. If at an early timepoint Chaserr homozygous embryos look 'normal' before they exhibit defects (see above). The authors should compare these embryos by

RNA-seq and not the MEFs – which are an artificial system and produce extracellular matrix and are not precursor tissues, for e.g. the bones. The random selection of tissues selected might be owed to the fact that the mice exhibit pleiotropic defects, the skeletal defects are striking, and focus should be put on tissues that are directly connected – such as limbs.
6) The authors go on and show that Chd2 protein is involved in regulating the expression of genes that are in tandem to another gene. They state an 'mild yet highly significant' effect. From the data they provide I am missing the analysis, how many expressed loci with that configuration are present and how many are depending on Chd2 protein? This analysis must be included or, if it in there somewhere, made clearer.

Minor comment:
1) The details of the Chd2m/m mutation have to described in more detail. Why has this allele no gross morphological defects? The allele the authors mention (Chd2 tm1b(EUCOMM)Hmgu has strong defects according to MGI – please check this and expand.
http://www.informatics.jax.org/allele/summary?markerId=MGI:2448567&alleleType=Targeted

Reviewer #2 (Remarks to the Author):

In this manuscript, Rom et al. describe the function of the long noncoding RNA (lncRNA) Chaserr, which is transcribed upstream of the chromatin remodeler Chd2. The authors generated Chaserr knockout (Cahserr-KO) mice for in vivo developmental studies. Deletion of the promoter and first exon of Chaserr abolished production of this lncRNA, and there was an increase in Chd2 expression. No Chaserr-KO mice were born, and mice with heterozygous deletion had reduced postnatal survival and growth retardation. The authors then generated Chaserr-KO (promoter and first exon deletion) in mice with a hypomorphic allele of Chd2 (Chd2m). Chaserr-KO;Chd2m/m mice were born in expected numbers, indicating that a hypomorphic allele of Chd2 can rescue the lethal phenotype of Chaserr-KO. In studies using Chaserr+/- Chd2m/+ mice, only the Chd2 allele from the same chromosome as the Chaserr-KO allele was upregulated, consistent with Chaserr regulating Chd2 expression in cis. The authors also studied Chaserr in cell culture. In mouse ESCs (as well as neurons from these ESCs), both Chaserr promoter-exon1 deletion and Chaserr "gene body" deletion resulted in increased levels of Chd2. Expression of Chaserr from dox-inducible vectors did not decrease this Chd2 overexpression. Then, the authors studied gene expression changes in mouse embryonic fibroblasts (MEFs) from crosses of Chaserr-KO and Chd2m/m mice. In this data, the authors noticed that the promoters of differentially expressed genes with Chaserr-KO were closer to the transcriptional termination site (TTS) of the closest upstream tandem gene. ATAC-seq of MEFs showed mild changes in promoter accessibility of genes differentially expressed with Chaserr-KO, and fewer changes were observed in Chaserr-KO;Chd2m/m MEFs. CHD2 MNase-ChIP-seq showed that CHD2 protein is found in the region around the TTS. From these analyses, the authors suggest that CHD2 normally functions in regions downstream of the TTS in WT cells, and that increased CHD2 levels from Chaserr-KO causes repression of promoters adjacent to those TTS regions.

There are currently only a handful of lncRNA studies that use genetic methods in vivo, and the main strength of this manuscript derives from its combination of several different genetic manipulations to investigate Chaserr function. Importantly, the authors provide in vivo developmental evidence that Chaserr functions by regulating Chd2 in cis. I particularly liked the rescue of Chaserr-KO by the Chd2 hypomorphic allele, as this result strongly suggests that the severe developmental phenotype of Chaserr-KO is mediated primarily by CHD2.

The part of the manuscript that relates to CHD2 function (the suggestion that CHD2 causes transcriptional interference of genes downstream of highly-expressed genes) was not very convincing and does not necessarily strengthen the manuscript. On some level, I think a mechanistic

understanding of CHD2 function may be beyond the scope of this paper (and so maybe this section could be removed). Instead, it might make more sense to focus more on the mechanistic relationship between Chaserr and Chd2. Some specific comments/suggestions follow:

1. While the Chaserr- “gene body” knockout left the promoter intact, was there any promoter activity at the Chaserr promoter region? From the data shown, it seems that the gene body knockout caused the absence of any Chaserr reads, suggesting that this large deletion disrupted Chaserr promoter activity. If this is the case, perhaps promoter competition between Chaserr and Chd2 is still a viable mechanistic model?

2. The authors note that poly-A insertion into Chaserr does not lead to upregulation of Chd2 (and this has been also reported by others). If poly-A insertion doesn’t cause a decrease in Chaserr promoter activity, and if promoter competition is a part of this lncRNA mechanism, perhaps this is why Chd2 wasn’t increased?

3. The Chaserr isoforms in mice could be better described, since the mouse Ref-seq annotation includes an isoform that overlaps with Chd2. Given the cis-regulatory role proposed, this could be an important point. RACE-cloning could be considered for this.

4. The authors should downplay their conclusions that are derived from a lack of correlation, or from comparisons between unmatched samples. For example:
a. “Hundreds of genes were dysregulated in the Chaserr–/– Chd2m/m mEFs compared to WT mEFs from the same pregnancies, and these changes are likely due to Chd2 loss-of-function, as there was no correlation between these changes and the changes in Chaserr–/– mEFs”. To support this claim, the authors could instead determine whether there is a correlation with Chd2m/m MEFs.
b. The authors find anti-correlation between RNA-seq data from Chaserr knockout embryos and Chd2+/- MGE, but this is difficult to interpret due to the different tissue types being compared.

5. In the Chaserr-Chd2-reporter plasmid transfection experiments, does ASO-mediated Chaserr knockdown also cause increased Chd2-luciferase activity? Since the Chaserr construct is from the cDNA, does this mean that splicing of the lncRNA is not necessary for the regulation of Chd2? Have the authors tried replacing Chaserr with a different sequence, to see if this local regulation is specific to Chaserr sequences?

6. As mentioned above, the mechanistic conclusions regarding CHD2 function are not as strong as the analysis of Chaserr developmental function and its cis-regulation of Chd2.
a. The authors propose that the overall mechanistic function of Chaserr is through its regulation of Chd2 expression, and that Chd2 overexpression upon loss of Chaserr leads to “increased transcriptional interference by inhibiting promoters found downstream of highly expressed genes”. However, they only describe 9 potential downstream genes that exhibit reduced core promoter accessibility.
b. While there are reads from CHD2 MNase-ChIP-seq in these 9 regions, it is unclear from the data presented whether this is above background levels.
c. The authors claim that “CHD2 preferentially associates with ~2Kb regions immediately downstream of TTSs”, however this isn’t obvious to me from the data presented.
d. In the analysis of the genome-wide ATAC-seq data, it does not seem that t-tests are the most appropriate statistical test in this context. Perhaps MACS would be more appropriate.

Point-by-point response to the reviewers for Rom et al.

Summary of the new experimental data in the revised manuscript
Following the suggestions and requests of the reviewers, we have added the following new data to the manuscript (alongside additional analyses described in detail below):

Characterization of the *Chaserr* transcript:

1. **Single molecule FISH** in mEFs supporting nuclear enrichment of *Chaserr* (Fig. 1b).
2. **3' RACE** supporting *Chaserr* 3' end annotation derived from RNA-seq and 3P-seq data (Extended Data Fig. 1b).

Characterization of *Chaserr* loss-of-function phenotype:

3. **Genotype distributions in E18.5 embryos from *Chaserr*$^{+/-}$ intercrosses**, showing that *Chaserr* is not essential for embryonic development (Fig. 1e).
4. **RNA-seq of *Chd2*$^{m/m}$ mEFs** showing inverse effects compared to *Chaserr*$^{-/-}$ mEFs (Fig. 4d).
5. **RNA-seq of E13.5 *Chaserr*$^{-/-}$ embryo** showing effects similar to these observed in *Chaserr*$^{-/-}$ mEFs (Fig. 4g).
6. **Pol2 ChIP-seq in *Chaserr*$^{-/-}$** mEFs showing that a substantial part of the changes in gene expressed observed in *Chaserr*$^{-/-}$ mEFs can be attributed to a decrease in Pol2 pausing (Fig. 6a-c).

Characterization of *Chaserr* mode of action:

7. **Knockdown of *Chaserr* in the compound heterozygous *Chaserr*$^{+/-}$ *Chd2*$^{m/+}$ cells**, showing that *Chaserr* knockdown up-regulates *Chd2* strictly in *cis* (Fig. 3e).
8. **4C analysis of *Chaserr*$^{p/p}$ and *Chaserr*$^{b/b}$ mESCs** showing that deletion of *Chaserr* promoter substantially affects the spatial interaction landscape of the *Chd2* promoter, but *Chaserr* gene body deletion does not lead to substantial changes in chromatin interaction (Fig. 2e-f).
9. **Pol2 ChIP-seq in *Chaserr*$^{b/b}$ mESCs** showing that in these cells, in which the gene body of *Chaserr* has been deleted, *Chaserr* promoter is still capable of efficiently recruiting Pol2 (Extended Data Fig. 2a).
10. **CHD2 RIP-seq** showing that CHD2 associates with hundreds of RNAs, including *Chaserr*, and that CHD2-bound RNAs tend to be up-regulated in *Chaserr*$^{-/-}$ and repressed in *Chd2*$^{m/m}$ mEFs (Fig. 6e-h).

Reviewer 1
The study by Rom and colleagues analyses the conserved lncRNA locus Chaserr, upstream of the Chd2 gene, which encodes a chromatin modifying protein essential for development and the human orthologue is implicated in human pathogenesis. The authors show that Chaserr's only function is to negatively regulate Chd2 expression levels. The authors characterize the effect from excess amount of Chd2 protein, but do not provide anything how Chaserr regulates Chd2; which I think is the burning question. Although Chaserr is an interesting locus, this work combines data from sometimes random samples and tissues. Why do the authors conclude that just because Chd2 is active in most tissue one

cannot stick to one particular tissue/cell type for analysis? It is well established that pleiotropic expressed genes still have distinct functions in specific cell types.
It remains completely unclear if the RNA is the carrier of the function or the transcriptional process; I see the latter to be major part. An interesting part of this work is that blocking the RNA of Chaserr could be of therapeutic potential to adjust Chd2 function in patients that have a functional inactive Chd2 allele. In that direction the RNA vs locus issue must be resolved.
If the following points are not addressed comprehensively, I cannot recommend publication in Nature Communications.

Major points:
1) The authors claim that Chaserr RNA is localized, or at least enriched, in the nucleus. From their data in Fig 1b this is hardly visible. Why do the authors analyze mouse duodenum, a tissue known to have a high autofluorescence activity? The data are not convincing and multiple spots in the nuclei are seen in Fig 1b. They isolated MEFs later in their study and the RNA FISH data must be repeated in an ex vivo cell type. Some convincing RNA FISH pictures are seen in Cabili, Genome Bology, 2016. In addition, the RNA FISH have to be correctly controlled by performing them in parallel in Chaserr null MEFs. These data must be complemented with a fractionation analysis of chromatin/nucleus/cytosol and not solely nucleus and cytosol. Again, it is important to refine this part for the subsequent points in this study -> while overexpression of Chaserr has no effect on Chd2 expression, LNA based Knock-down has an effect. It must be clearly shown that Chaserr RNA resides at is locus and eventually at its origin of production.

Following the request of the reviewer, we have now performed single-molecule FISH with probes targeting *Chaserr* exons or introns in WT and *Chaserr*$^{-/-}$ mEFs, which shows that *Chaserr* is indeed localized to its site of transcription in these cells. Further, we show using available fractionation RNA-seq data from mESCs that *Chaserr* is as enriched in the chromatin fraction as known chromatin-associated lncRNAs such as *MALAT1* and *NEAT1*. These data are shown in **Figure 1b** and **Extended data Figure 1c**:

b

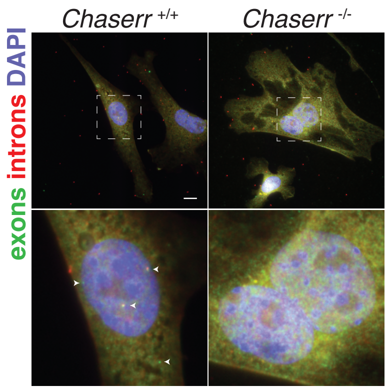

Fig. 1b Single-molecule FISH with probes targeting *Chaserr* introns (red) and exons (green) in mouse embryonic fibroblasts. *Chaserr* exons are marked by white arrowhead. Scale bar shows 10µm.

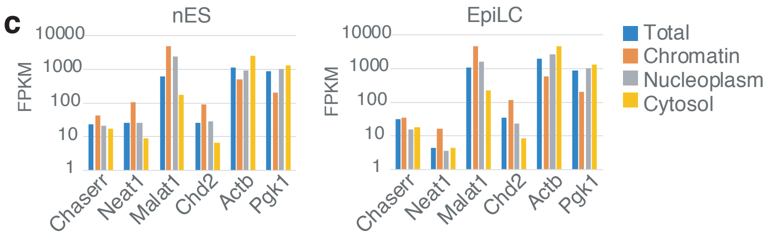

Extended Data Fig. 1c Expression levels of the indicated RNAs in the indicated compartment of naïve embryonic stem cells (nES) or epiblast-like cells (EpiLCs) (taken from GEO GSE99366).

2) Following the previous point: If exogenous overexpression of Chaserr has no effect, CRISPR-i guided overexpression of the endogenous locus can be performed. This would also clarify the point of RNA production at its endogenous locus is regulating Chd2. If the authors tried this without success: either show the data or try it in a different cell type than ESCs. A minimum of two different cell types must be tested before one can conclude that CRISPR-i overexpression doesn't work on this locus.

We thank the reviewer for this suggestion. We have now used CRIPSRa in Neuro2a cells with five gRNAs which target the *Chaserr* promoter region, which resulted, as predicted by our model, in an increase in *Chaserr* expression, and a concomitant decrease in the expression of *Chd2*.

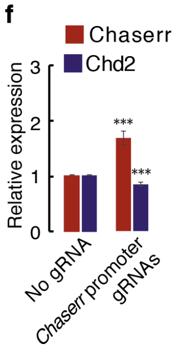

Fig. 2f qRT-PCR of the indicated genes in the indicated backgrounds or treatments. Normalized to *Actb*. n=5. Error bars show S.E.M. ***P<0.001 (two-sided t-test).

Page 5: "Furthermore, induction of *Chaserr* expression from the endogenous locus using CRISPR activation[28] resulted in a decrease in *Chd2* mRNA levels (**Fig. 2f**)."

We note that the increase in *Chaserr* expression that we are able to get in this system, using five gRNAs, is relatively modest (~65% over WT levels), likely because the promoter is already considerably active, and accordingly the decrease in *Chd2* levels is ~20%, which is nevertheless highly significant ($P=1.5\times10^{-4}$).

3) In the initial Chaserr mutant analysis the author show that they never find homozygous pubs. At what stage are the embryos showing first defects? The authors have to determine at least the timepoint when the Chaserr homozygous embryos are dying. Later the show that homozygous ESCs are viable, so some development should occur. See more below point 5.

Following the request of the reviewer, we now obtained statistics of the genotypes of embryos from *Chaserr*$^{+/-}$ intercrosses at E18.5, which are now shown in **Fig. 1e**:

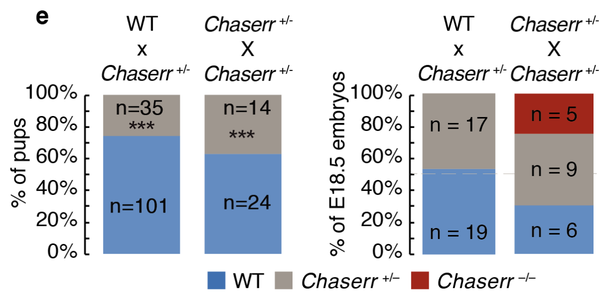

Fig. 1e Left: Neonate survival rates for the indicated crossing. Right: Embryonic survival rates at the indicated embryonic time points.

These do not appear to deviate from Mendelian ratios, and so we conclude that *Chaserr* is not required for embryonic survival:

Page 4: "In contrast, embryos were recovered with the expected Mendelian ratios in crosses between *Chaserr*$^{+/-}$ and *Chaserr*$^{+/+}$ mice and in *Chaserr*$^{+/-}$ intercrosses (**Fig. 1e** and **Extended Data Fig. 1l**), suggesting that *Chaserr* is required for postnatal, but not embryonic, survival."

As it is exceptionally difficult to get *Chaserr*$^{+/-}$ mice pregnant (see point 5 below), further narrowing down of the time window at which *Chaserr*$^{-/-}$ pups are dying, and their cause of death, would take many months, and we believe that it is out of scope of the current study.

4) The cis-acting activity in the heterozygous compound mutant Chaserr/Chd2 hints towards a Chaserr RNA independent function. Therefore, the lncRNA locus rather than the RNA itself is active. In that direction the title is misleading and must be changed: the lncRNA locus regulates Chd2 not the RNA.

Following the request of the reviewer we revised the title of the manuscript to "Regulation of CHD2 expression by the Chaserr long noncoding RNA gene is essential for viability".

I see strong similarities with the PVT1 locus work (Cho at al., Cell, 2018) from the Chang lab, where they show a function of the lncRNA PVT1 promotor on the cis gene expression. The authors provide ExtFig4 (why adult mouse brain?) that only shows two major domains: 1) the super-enhancer region upstream of Chaserr and the tight interaction of the Chaserr/Chd2 region. Eventually, Chaserr is the 'border' and by deleting Chd2 promoter (again: see PVT1) they collapsed this separation and 3C analysis could show this.

We agree with the reviewer that there are some similarities between *Chaserr*/*Chd2* and *Pvt1*/Myc, but also note that there is a key difference between the observations about these loci, which we think strongly argues that a different mechanism is in play – knockdown of *PVT1* using ASOs does not affect *MYC* (Fig. 3B in Cho et al.), whereas knockdown of *Chaserr* results in an increase in *Chd2* mRNA and protein levels (**Fig. 2f**). Also, deletion of the gene body of *Chaserr*, in the *Chaserr*$^{b/b}$ mESCs, does not remove the *Chaserr* promoter, which remains enriched for Pol2 in ChIP-seq data (**Extended Data Fig. 2a**, see below), yet leads to increases in *Chd2* levels. In the revised manuscript, we further strengthen this point by performing *Chaserr* GapmeR-mediated knockdown in *Chaserr*$^{+/-}$ *Chd2*$^{m/+}$ compound heterozygous mEFs. These cells express *Chaserr* only from the allele in cis to *Chd2*^{m}, and allele-specific PCR allows us to test which allele of *Chd2* is affected by the knockdown. We found that *Chaserr* knockdown results in up-regulation of only *Chd2*^{m} (**Fig. 3e**), further solidifying the model in which *Chaserr* production or the RNA product are required for repression of *Chd2*:

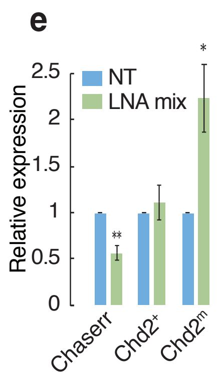

Fig. 3e Changes in expression of *Chaserr*, or of *Chd2* mRNA from the indicated allele, in *Chaserr*$^{+/-}$ *Chd2*$^{m/+}$ mEFs transfected with a mix of two LNAs targeting *Chaserr* or non-targeting control (NT).

Page 7: "mEFs derived from E13.5 *Chaserr*$^{+/-}$ *Chd2*$^{m/+}$ embryos allowed us to test whether the effect of knocking down *Chaserr* on *Chd2* also occurs strictly in *cis*. Indeed, treatment of these mEFs with LNA Gapmers targeting *Chaserr* (which is expressed only from the *Chd*^{m} allele) resulted in an increase of expression of *Chd*^{m} mRNA, but did not affect *Chd*$^{+}$ mRNA levels (**Fig. 3e**), further solidifying the *cis*-acting function of *Chaserr* RNA product or the act of its transcription."

We now also show that the landscape of spatial proximities as captured by HiC is very similar in the mouse brain and in mESC cells (**Extended Data Fig. S5**):

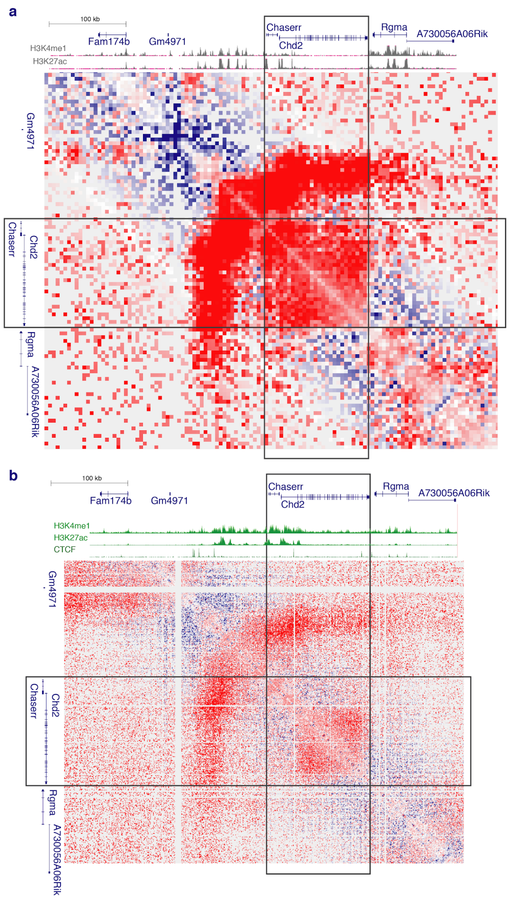

Extended Data Figure 5 | Spatial contacts in the *Chaserr/Chd2* locus. Hi-C data from adult mouse brain[83] (**a**) and mESCs[84] (**b**) visualized using JuiceBox[85]. Regions with excess of contacts over expected are in red and those with depletion of contacts are in blue. The box surrounds the *Chaserr*/*Chd2* locus. H3K4me1 and H3K27ac chromatin marks and CTCF ChIP-seq tracks are from the ENCODE project data for mouse whole brain (a) and mESCs (b).

To further study how perturbations of the *Chaserr* locus affect the spatial contact landscape of the *Chd2* promoter we now used 4C with *Chd2* promoter as a viewpoint in WT, *Chaserr* promoter deletion (*Chaserr*$^{p/p}$) and Chaserr gene body deletion (*Chaserr*$^{b/b}$) mESCs. We find that, as could be expected, *Chaserr* promoter deletion increases the contacts of the *Chd2* promoter with the shared enhancers

found upstream of the *Chaserr/Chd2* locus. However, *Chaserr* gene body deletion, which leads to about as much up-regulation of Chd2, has a mild effect, if any, on the contact landscape (**Fig. 2g**). We conclude that while promoter competition may contribute to the increase in Chd2 levels in *Chaserr*$^{-/-}$ cells, the dominant contribution is of transcription through the *Chaserr* locus or its RNA product. We elaborate on this topic in the Results and Discussion sections.

Page 5: "Importantly, *Chaserr*$^{b/b}$ mESCs have an intact *Chaserr* promoter, which recruits RNA Pol2 (**Extended Data Fig. 2a**), and thus this line helps address the possibility of a competition between the *Chaserr* and *Chd2* promoters over shared enhancers. Indeed, Targeted Chromosome Conformation Capture (4C) analysis of the two lines showed that while deletion of *Chaserr* promoter resulted in a pronounced increase of contacts between the *Chd2* promoter and an array of enhancers upstream of the *Chaserr*/*Chd2* locus, no substantial changes were observed in the *Chaserr*$^{b/b}$ cells (**Fig. 2g**), suggesting that enhancer competition is not the predominant cause of the increased *Chd2* levels in these cell lines."

Discussion Page 12: "Another possibility is that the promoters of *Chaserr* and of *Chd2* compete for binding to shared enhancer elements, and elimination of the *Chaserr* promoter increases the ability of these enhancers to boost Chd2 expression, as recently described for *MYC* and *PVT1*[52]. Both *Chaserr* and *Chd2* appear to be under the shared control of several enhancer regions found in the ~200 Kb gene desert upstream of *Chaserr* (**Fig. 2g** and **Extended Data Fig. 5**), and those enhancers potentially regulate both genes together, which can explain the tight co-expression between *Chaserr* and *Chd2* mRNA (**Fig. 1c**). Indeed, deletion of the *Chaserr* promoter in mESCs leads to an increase in contacts between the *Chd2* promoter and three broad upstream enhancer regions (**Fig. 2g**). Enhancer competition may therefore contribute to the increase in *Chd2* expression in *Chaserr*$^{-/-}$ cells. However, it is unlikely that such competition explains much of the CHD2 up-regulation, as we observe similar levels of up-regulation when using ASO Gapmers in different cellular systems (**Fig. 2e**, **Fig. 3e**, **Extended Data Fig. 3b-c**, and see below). In addition, removing just the gene body of *Chaserr*, which leaves the promoter intact, does not substantially affect Pol2 occupancy at the promoter (**Extended Data Fig. 2a**), nor the landscape of spatial contacts between the *Chd2* promoter and the upstream enhancers (**Fig. 2g**), despite leading to a similar upregulation of *Chd2* levels (**Fig. 2e**)."

5) The RNA-seq analysis in Fig 4 is confusing and not straight forward; the authors compare expression profiles of random tissues, cells and structures. Why do the authors only compare het embryos and not homozygous? Please explain. If at an early timepoint Chaserr homozygous embryos look 'normal' before they exhibit defects (see above). The authors should compare these embryos by RNA-seq and not the MEFs – which are an artificial system and produce extracellular matrix and are not precursor tissues, for e.g. the bones. The random selection of tissues selected might be owed to the fact that the mice exhibit pleiotropic defects, the skeletal defects are striking, and focus should be put on tissues that are directly connected – such as limbs.

We agree with the reviewer that extensive analysis of *Chaserr*$^{-/-}$ embryos, including specific tissues such as limbs, would be ideal. However, *Chaserr*$^{+/-}$ females get pregnant very rarely - in three years of working with these mice, we observed <30 pregnancies while using at least 10 timed pregnancy breeding cages (and these pregnancies were used in part for the genotyping statistics of *Chaserr*$^{-/-}$ pups

and now also E18.5 embryos). We believe the compromised fertility of *Chaserr*$^{+/–}$ mice might be due to ovarian defects, but these are out of scope of the current study. Our breeding capabilities were further constrained by the overall limited numbers of *Chaserr*$^{+/–}$ females surviving to adulthood. Therefore, we prefer to focus on mEFs, which allowed us to efficiently apply multiple experimental modalities (RNA-seq, ATAC-seq, FISH, and now also Pol2 ChIP-seq) on a relatively homogenous cell populations. To illustrate that transcriptional dysregulation observed in *Chaserr*$^{–/–}$ mEFs is physiologically relevant, we now performed RNA-seq on a *Chaserr*$^{–/–}$ E13.5 embryo, and found transcriptional changes that mirror those observed in *Chaserr*$^{–/–}$ mEFs (Spearman R=0.4, $P<10^{-15}$; **Fig. 4g**). With these new data, as well as RNA-seq from *Chd2*$^{m/m}$ mEFs, we now rearranged and focused **Fig. 4** on *Chaserr*$^{–/–}$ cells, and moved the analysis of the heterozygous embryos to the **Extended Data Fig. 4**.

6) The authors go on and show that Chd2 protein is involved in regulating the expression of genes that are in tandem to another gene. They state an 'mild yet highly significant' effect. From the data they provide I am missing the analysis, how many expressed loci with that configuration are present and how many are depending on Chd2 protein? This analysis must be included or, if it in there somewhere, made clearer.

We apologize for not stating this case with sufficient clarity. Following this request, and the comments of reviewer #2 (see below) we now revised the RNA-seq and the ATAC-seq analysis (**Fig. 4** and **5**). We now improved the description of the changes observed in ATAC-seq and in the ATAC-seq data, and show that over one hundred show evidence of repression via transcription interference:

Page 9: "Comparing WT and *Chaserr*$^{–/–}$ mEFs, we found 80 and 677 regions with increased or reduced accessibility, respectively (DESeq2 P<0.05), with 6 induced and 24 reduced peaks occurring near TSSs. Promoter peaks whose accessibility was significantly decreased in *Chaserr*$^{–/–}$ mEFs were associated with repressed genes in the RNA-seq data (**Fig. 5a**). Strikingly, these promoters were separated by short intergenic regions from TTSs of other genes on the same strand (**Fig. 5a-b**, median distance of 3.3 Kb compared to 43.8 Kb for unaffected promoters). Notably, the upstream genes were expressed at dramatically higher levels than the affected genes or other genes (17-fold difference between median expression, **Fig. 5a**), and their expression was not substantially affected by loss of *Chaserr* (**Fig. 5a**). Promoters of 96 additional genes which showed reduction of >25% in *Chaserr*$^{–/–}$ mEFs in the RNA-seq data and had an upstream neighbor on the same strand within <2 Kb, had reduced ATAC-seq signal in *Chaserr*$^{–/–}$ mEFs when considered as a group (P=0.014, Wilcoxon two-sided test). Altogether, transcriptional interference from highly expressed and close upstream neighbors reduces promoter accessibility and gene expression over one hundred genes in *Chaserr*$^{–/–}$ mEFs."

Minor comment:
1) The details of the Chd2m/m mutation have to described in more detail. Why has this allele no gross morphological defects? The allele the authors mention (Chd2 tm1b(EUCOMM)Hmgu has strong defects according to MGI – please check this and expand.
http://www.informatics.jax.org/allele/summary?markerId=MGI:2448567&alleleType=Targeted

Our observations in the Chd2$^{m/m}$ mice are largely consistent with those of mice homozygous for the Chd2$^{tm1b(EUCOMM)Hmgu}$ allele (https://www.mousephenotype.org/data/genes/MGI:2448567) in that mice are viable, fertile, don't have severe phenotypic manifestations (e.g, EUCOMM mice are 7 mm shorter than WT and their tibia are ~1.5mm shorter than WT). We did observe a reduction in size and subcutaneous fat in the *Chd2*$^{m/m}$ mice, but did not systematically measure all the parameters described for *Chd2*$^{tm1b(EUCOMM)Hmgu}$ in our Chd2$^{m/m}$ mice as our cohort is smaller and this is not the focus of our study. We altered the text to reflect this:

Page 6: "*Chd2*$^{m/m}$ mice were born at expected Mendelian ratios (**Extended Data Fig. 2e**), and similarly to the *Chd2*$^{tm1b(EUCOMM)Hmgu}$ mice generated by insertion of a LacZ cassette into the 2nd intron and deletion of 3rd exon of *Chd2*[30], had mild reduction in size and in subcutaneous adipose tissue (not shown), but no gross developmental or fertility phenotypes."

Reviewer 2

In this manuscript, Rom et al. describe the function of the long noncoding RNA (lncRNA) Chaserr, which is transcribed upstream of the chromatin remodeler Chd2. The authors generated Chaserr knockout (Cahserr-KO) mice for in vivo developmental studies. Deletion of the promoter and first exon of Chaserr abolished production of this lncRNA, and there was an increase in Chd2 expression. No Chaserr-KO mice were born, and mice with heterozygous deletion had reduced postnatal survival and growth retardation. The authors then generated Chaserr-KO (promoter and first exon deletion) in mice with a hypomorphic allele of Chd2 (Chd2m). Chaserr-KO;Chd2m/m mice were born in expected numbers, indicating that a hypomorphic allele of Chd2 can rescue the lethal phenotype of Chaserr-KO. In studies using Chaserr+/- Chd2m/+ mice, only the Chd2 allele from the same chromosome as the Chaserr-KO allele was upregulated, consistent with Chaserr regulating Chd2 expression in cis. The authors also studied Chaserr in cell culture. In mouse ESCs (as well as neurons from these ESCs), both Chaserr promoter-exon1 deletion and Chaserr "gene body" deletion resulted in increased levels of Chd2. Expression of Chaserr from dox-inducible vectors did not decrease this Chd2 overexpression. Then, the authors studied gene expression changes in mouse embryonic fibroblasts (MEFs) from crosses of Chaserr-KO and Chd2m/m mice. In this data, the authors noticed that the promoters of differentially expressed genes with Chaserr-KO were closer to the transcriptional termination site (TTS) of the closest upstream tandem gene. ATAC-seq of MEFs showed mild changes in promoter accessibility of genes differentially expressed with Chaserr-KO, and fewer changes were observed in Chaserr-KO;Chd2m/m MEFs. CHD2 MNase-ChIP-seq showed that CHD2 protein is found in the region around the TTS. From these analyses, the authors suggest that CHD2 normally functions in regions downstream of the TTS in WT cells, and that increased CHD2 levels from Chaserr-KO causes repression of promoters adjacent to those TTS regions.

There are currently only a handful of lncRNA studies that use genetic methods in vivo, and the main strength of this manuscript derives from its combination of several different genetic manipulations to investigate Chaserr function. Importantly, the authors provide in vivo developmental evidence that Chaserr functions by regulating Chd2 in cis. I particularly liked the rescue of Chaserr-KO by the Chd2

hypomorphic allele, as this result strongly suggests that the severe developmental phenotype of Chaserr-KO is mediated primarily by CHD2.

We thank the reviewer for a positive evaluation of our work.

The part of the manuscript that relates to CHD2 function (the suggestion that CHD2 causes transcriptional interference of genes downstream of highly-expressed genes) was not very convincing and does not necessarily strengthen the manuscript. On some level, I think a mechanistic understanding of CHD2 function may be beyond the scope of this paper (and so maybe this section could be removed). Instead, it might make more sense to focus more on the mechanistic relationship between Chaserr and Chd2.

We agree with the reviewer that understanding the *Chaserr* mode-of-action is of high interest, and we have indeed invested substantial efforts in it. We believe that *Chaserr* biology is tightly connected to the largely unknown functions of CHD2. To further illustrate this point, and strengthen the evidence for a feedback loop involving Chaserr and CHD2, we now obtained RNA-seq data of *Chd2*$^{m/m}$ mEFs that show a reduction in *Chaserr* levels alongside an increase in *Chd2* mRNA, and RIP-seq of CHD2, that shows that *Chaserr* is one of the RNAs most significantly associated with CHD2:

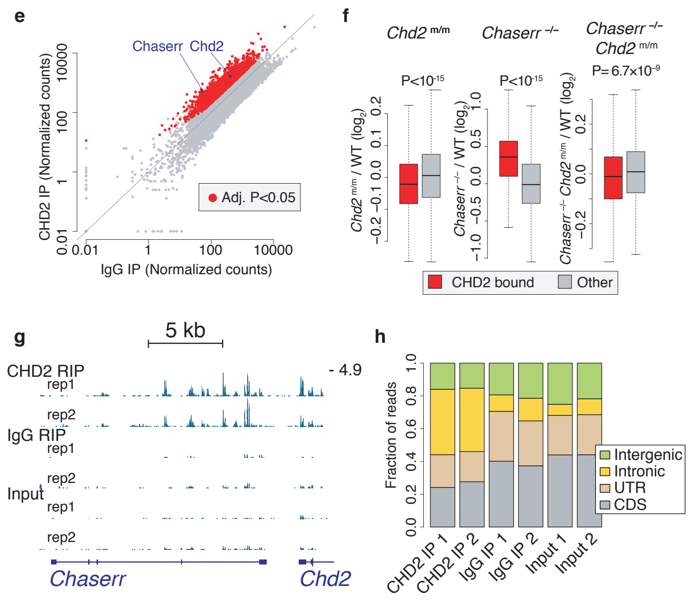

Fig. 6e Normalized RIP-seq read counts within gene bodies in CHD2 and IgG RIP libraries (average of two replicates). Red dots correspond to genes with adjusted P<0.05 (DESeq2 analysis). **f,** Changes in gene expression of genes significantly associated with CHD2 (from e) and other gene expressed in mEFs in the indicated RNA-seq-based comparison. **g,** RIP-seq read coverage in the *Chaserr* locus. **h,** Fractions of reads mapping to the indicated regions in the indicated RIP-seq library.

In the Discussion we now mention that the CHD2-Chaserr interaction supports the possibility of a feedback loop involving the two genes:

Page 11: "Due to the short intergenic region between *Chaserr* and *Chd2*, we suggest that such transcriptional interference serves as the basis of a negative feedback loop that leads to repression of *Chd2* production when CHD2 protein levels are high, thus maintaining a tight control on its expression. In cells with *Chaserr* loss-of-function, this loop is compromised, and CHD2 is upregulated. Supporting this model, in *Chaserr*$^{+/-}$ *Chd2*$^{m/+}$ tissues, increase in CHD2 protein leads to repression of transcription of

the *Chd2*^{m} allele, which is found next to an intact *Chaserr* (**Fig. 3d**). Furthermore, reduction in CHD2 protein in *Chd2*$^{m/m}$ mEFs leads to a decrease in *Chaserr* levels and an increase in *Chd2* mRNA levels (**Fig. 4d**). Lastly, we found that CHD2 protein associates with the *Chaserr* RNA (**Fig. 6e**), supporting the possibility that excess CHD2 levels can be sensed by *Chaserr*."

We believe that this and additional analyses described above and below strengthen the part of the manuscript that pertains to the transcriptional dysregulation following CHD2 over-expression that results from loss of *Chaserr*.

Some specific comments/suggestions follow:

1. While the Chaserr- "gene body" knockout left the promoter intact, was there any promoter activity at the Chaserr promoter region? From the data shown, it seems that the gene body knockout caused the absence of any Chaserr reads, suggesting that this large deletion disrupted Chaserr promoter activity. If this is the case, perhaps promoter competition between Chaserr and Chd2 is still a viable mechanistic model?

Following this concern we now performed Pol2 ChIP-seq in an mESC line where the gene body of Chaserr is deleted (*Chaserr*$^{b/b}$), and show *Chaserr* promoter still efficiently recruits Pol2 (**Extended Data Fig. 2a**):

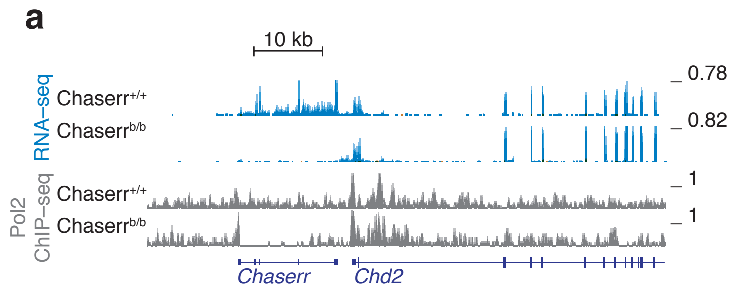

Extended Data Fig. 2a, RNA-seq and Pol2 ChIP-seq read coverage in the indicated mESC lines.

As mentioned above, we also performed 4C on these lines, and found that, unlike the *Chaserr* promoter deletion, *Chaserr* gene body deletion does not markedly affect the spatial contact landscape of the *Chd2* promoter, while still leading to *Chd2* induction (**Fig. 2e**).

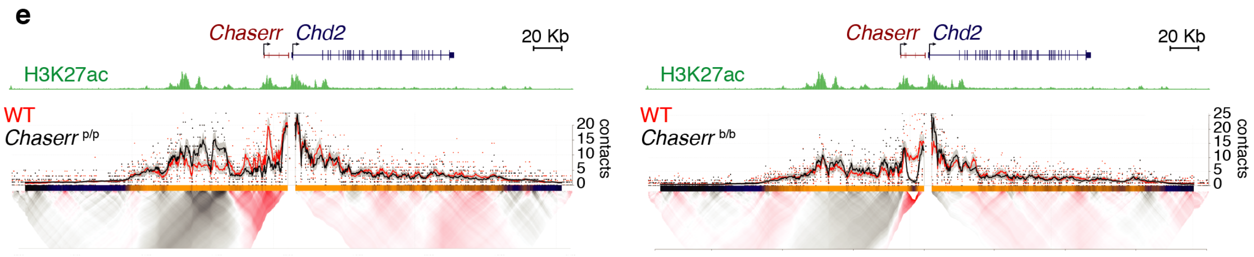

Fig. 2g Targeted Chromosome Conformation Capture (4C) analysis using the *Chd2* promoter as the viewpoint in WT vs. *Chaserr*$^{p/p}$ (left) or WT vs. *Chaserr*$^{b/b}$ (right) mESCs . Top to bottom: scheme of the *Chd2-Chaserr* locus; H3K27ac ChIP-seq signal in mESCs; smoothed trend lines and raw counts of the

contact profiles in the indicated cell lines; and domainogram[79] showing mean contact per fragment end for a series of window sizes.

2. The authors note that poly-A insertion into Chaserr does not lead to upregulation of Chd2 (and this has been also reported by others). If poly-A insertion doesn't cause a decrease in Chaserr promoter activity, and if promoter competition is a part of this lncRNA mechanism, perhaps this is why Chd2 wasn't increased?

Engreitz et al. attempted introduction of a triple poly(A) sequence into the *Chaserr* locus, and observed that it was not effective in reducing Chaserr levels below 40% of the WT levels ("data not shown" mentioned in Supplementary note 6 in Engreitz et al.). As a result, this model appears to be not useful for checking effects of *Chaserr* transcription on *Chd2* levels (from Supp. note 6 in Engreitz et al: *"Because we did not fully eliminate transcription in the linc2025 locus or expression of the linc2025 RNA, we cannot definitively assess the mechanism by which deleting the linc2025 promoter affects Chd2 expression"*)

3. The Chaserr isoforms in mice could be better described, since the mouse Ref-seq annotation includes an isoform that overlaps with Chd2. Given the cis-regulatory role proposed, this could be an important point. RACE-cloning could be considered for this.

As requested, we performed 3' RACE, and sequenced 10 clones that all support the 3' end derived from the RNA-seq and 3' seq data. 3' RACE clones are now shown in **Extended Data Fig. 1b**:

b

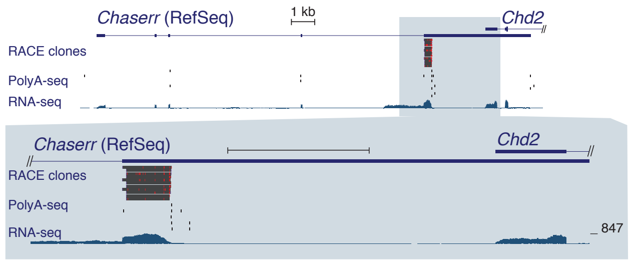

Extended Data Fig. 1b 3' RACE on RNA extracted from mEFs. Shown are BLAT alignments of Sanger sequencing of ten independent clones, alongside mEF RNA-seq and PolyA-seq clusters from[80].

4. The authors should downplay their conclusions that are derived from a lack of correlation, or from comparisons between unmatched samples. For example:

a. "Hundreds of genes were dysregulated in the Chaserr–/– Chd2m/m mEFs compared to WT mEFs from the same pregnancies, and these changes are likely due to Chd2 loss-of-function, as there was no correlation between these changes and the changes in Chaserr–/– mEFs". To support this claim, the authors could instead determine whether there is a correlation with Chd2m/m MEFs.

Following the suggestion of the reviewer, we now performed RNA-seq in *Chd2*$^{m/m}$ mEFs and WT littermates. These mEFs exhibit transcriptional changes that are significantly anti-correlated with those observed in *Chaserr*$^{-/-}$ and concordant with those observed in *Chaserr*$^{-/-}$ *Chd2*$^{m/m}$.

Nevertheless, since each of the genotypes is compared to WT littermates the data should indeed be interpreted with caution, and we now note this in the text:

Page 8: "Gene expression changes in *Chaserr*$^{-/-}$ mEFs were significantly inversely correlated with those in *Chd2*$^{m/m}$ mEFs, where *Chaserr* was down-regulated and *Chd2* was up-regulated (**Fig. 4d** and **Extended Data Fig. 4b)**. We also profiled *Chaserr*$^{-/-}$ *Chd2*$^{m/m}$ mEFs, and found no correlation between the changes observed between these and WT mEFs, and the changes in *Chaserr*$^{-/-}$ mEFs (Spearman R=-0.003 P=0.68). Instead, changes in *Chaserr*$^{-/-}$ *Chd2*$^{m/m}$ mEFs were similar to those in *Chd2*$^{m/m}$ mEFs (**Extended Data Fig. 4b**). The correlations between expression changes should be interpreted with caution as they are derived from different crosses, and yet they support the notion that the downstream effects of loss of *Chaserr* are driven by CHD2 up-regulation."

b. The authors find anti-correlation between RNA-seq data from Chaserr knockout embryos and Chd2+/- MGE, but this is difficult to interpret due to the different tissue types being compared.

We agree with the reviewer, and since we now have a much better comparison using *Chd2*$^{m/m}$ mEFs, we now removed the comparison with *Chd2*$^{+/-}$ MGE from the text.

5. In the Chaserr-Chd2-reporter plasmid transfection experiments, does ASO-mediated Chaserr knockdown also cause increased Chd2-luciferase activity? Since the Chaserr construct is from the cDNA, does this mean that splicing of the lncRNA is not necessary for the regulation of Chd2? Have the authors tried replacing Chaserr with a different sequence, to see if this local regulation is specific to Chaserr sequences?

In preparation of the revised manuscript, we have performed additional experiments using the luciferase reporter, and found that it was not as robust and reproducible as we initially perceived. Beyond the increase in luciferase levels from the plasmid that does not have an intact *Chaserr* promoter, the reporter did not mirror well the observations from the endogenous locus, possibly because of the lack of *Chaserr* introns, or because of lack of an internal control on the plasmid. We therefore decided to omit from the manuscript this part and focus on perturbations of the endogenous transcript.

6. As mentioned above, the mechanistic conclusions regarding CHD2 function are not as strong as the analysis of Chaserr developmental function and its cis-regulation of Chd2.

a. The authors propose that the overall mechanistic function of Chaserr is through its regulation of Chd2 expression, and that Chd2 overexpression upon loss of Chaserr leads to "increased transcriptional interference by inhibiting promoters found downstream of highly expressed genes". However, they only describe 9 potential downstream genes that exhibit reduced core promoter accessibility.

As mentioned above in response to reviewer #1, we now revised the ATAC-seq analysis and show that >100 genes with close upstream neighbors have evidence of both reduction in promoter accessibility and reduction of gene expression, and so transcription interference driven by CHD2 hyperactivity has a substantial impact.

In order to better understand the transcriptional consequences of CHD2 over-production mediated by loss of *Chaserr*, we now also performed Pol2 ChIP-seq in WT and *Chaserr*$^{-/-}$ mEFs. We found that significant changes in expression of 400 genes following loss of *Chaserr* could be explained by changes in Pol2 pausing, as now described in the text:

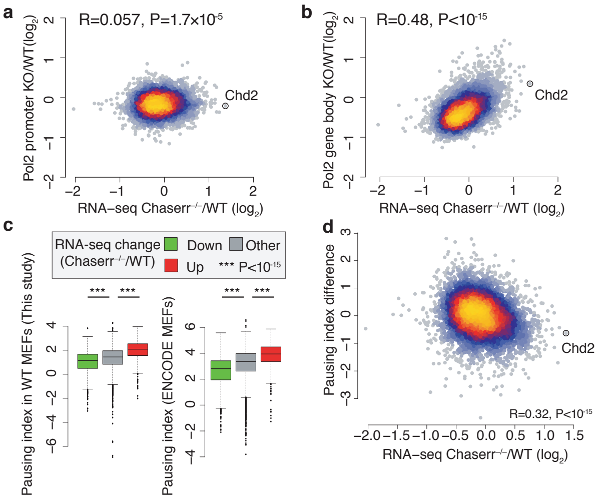

Fig. 6a Correlation between changes in gene expression levels and changes in Pol2 occupancy at the promoter region (±300 bases around TSS). **b,** As in a, but for changes in Pol2 occupancy at gene body regions (defined from 1 Kb downstream of the TSS to the TTS). **c,** Pol2 pausing index (see Methods) in our WT mEF Pol2 ChIP-seq data (left) and ENCODE mEF data (right) for genes with the indicated changes in gene expression (adjusted P<0.05). **d,** Changes in Pol2 pausing index as a function of changes in gene expression between *Chaserr*$^{-/-}$ and WT mEFs.

Page 10: "An additional underlying factor contributing to dysregulation of gene expression emerged from ChIP-seq[33] analysis of Pol2 in *Chaserr*$^{-/-}$ and WT mEFs. We observed a significant correlation between changes in gene expression and changes in Pol2 occupancy at gene bodies, but not at promoters (**Fig. 6a-b**). This observation, together with the known roles of CHD1 in regulation of Pol2 pausing[34], led us to suspect that Pol2 pausing might be affected by the increase in CHD2 levels caused by *Chaserr* loss. Indeed, genes up-regulated in *Chaserr*$^{-/-}$ mEFs were associated with a substantially higher pausing index in WT mEFs in our Pol2 ChIP-seq data, as well as in mEFs profiled by the ENCODE project (**Fig. 6c**); generally, increases in gene expression were correlated with reduction in Pol2 pausing at the affected genes (**Fig. 6d**). Overall, 400 (15.3%) of the genes significantly dysregulated in *Chaserr*$^{-/-}$ mEFs were associated with a corresponding change of >25% in Pol2 pausing."

b. While there are reads from CHD2 MNase-ChIP-seq in these 9 regions, it is unclear from the data presented whether this is above background levels.

We now analyzed significance of CHD2 occupancy downstream of TTSs genome-wide using Homer/DESeq2 and show that many genes highly expressed in mESCs have significant CHD2 occupancy in the region downstream of their TTS (**Fig. 5e**):

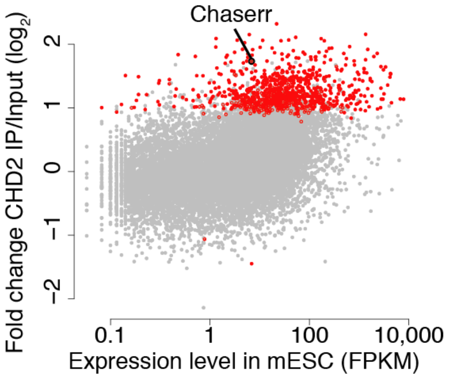

Fig. 5e. Enrichment of CHD2 occupancy in mESCs in the 2 Kb region downstream of the TTS when compared to input, as a function of mESC gene expression levels.

The enrichment of CHD2 occupancy in mESCs is significant in some of the examples we previously showed to be affected in mEFs, but not in others, possibly due to differences between mESCs and mEFs. we now removed the CHD2 ChIP-seq data from the panel focusing on specific genes (**Fig. 5b**), and revised the text according to the results of the new analysis:

Page 9: "Using tagged CHD2 MNase-ChIP-seq data from mESCs[13], we observed significant occupancy of CHD2 protein in the regions downstream of TTSs of hundreds of highly expressed genes, and downstream of *Chaserr* TTS (**Fig. 5d-e**), suggesting that the effect seen on the accessibility of the promoters and gene expression of genes found closely downstream of a TTS might be a direct result of increased CHD2 activity in intergenic regions."

c. The authors claim that "CHD2 preferentially associates with ~2Kb regions immediately downstream of TTSs", however this isn't obvious to me from the data presented.

We added a new analysis (**Figure 5f** above) that uses Homer/DESeq2 to compare the occupancy of CHD2 and input control in the 2Kb regions downstream of TTSs, and show that there is significant CHD2 occupancy downstream of ~800 highly expressed genes, including downstream of *Chaserr*. In addition, we now highlight the relevant region (TTS to 2kb downstream of the TTS) in the metagene plot in **Figure 5d**:

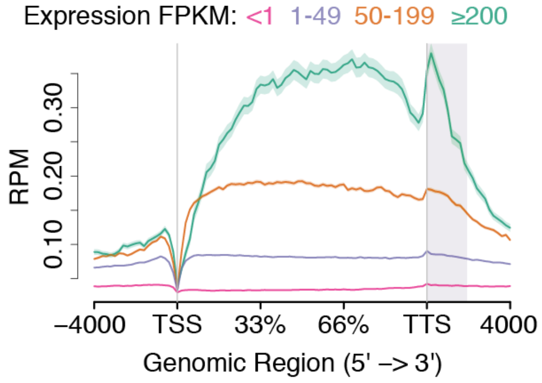

Fig. 5d Metagene of CHD2 occupancy[13] on genes in mESCs, divided into four groups based on their expression FPKM.

d. In the analysis of the genome-wide ATAC-seq data, it does not seem that t-tests are the most appropriate statistical test in this context. Perhaps MACS would be more appropriate.

Following the request of the reviewer, we have now used Homer/DESeq2 for the analysis of ATAC-seq and ChIP-seq data.

Reviewers' Comments:

Reviewer #1:
Remarks to the Author:
I want to congratulate the authors on this revised version of the manuscript. They addressed all my concerns and, in my opinion, the concerns from the other reviewer very carefully. The additional data they provide significantly improves the manuscript. The authors provide now more data on the regulation of Chd2 by the lncRNA gene Chaserr. Although they cannot provide the full mechanism, they extend their analysis in the best possible way. It will be challenging in the future how lncRNA gene regulate their neighboring genes; might it be the transcriptional process, the RNA or both. I highly recommend publication in Nature Comm.

Reviewer #2:
Remarks to the Author:
In this revised manuscript, Ulitsky and colleagues have performed new experiments and analyses to address my major comments. The new 4C analysis nicely demonstrates that Chaserr gene body deletion does not substantially change the spatial contact landscape of the local chromosome (while the Chaserr promoter deletion does cause changes). Also, the Chaserr gene body deletion still can recruit Pol2 (new data). These data (along with the new 3'RACE to confirm that most Chaserr transcripts terminate before Chd2) strengthen the mechanistic understanding of this lncRNA. Finally, the authors have identified about 100 genes with close upstream neighbors that have changes in promoter accessibility and decreased gene expression, bolstering the part of their story about CHD2 function. In my opinion, this manuscript is now suitable for publication.
